# Brain–Computer Interfaces: The Dawn of a New Era in Disease Treatment

**DOI:** 10.1002/EXP.20250452

**Published:** 2026-05-28

**Authors:** Yuqi Feng, Wangzheqi Zhang, Jun Chen, Huang Wu, Lei Wu, Yanhao Qiu, Xiaoming Deng, Chenglong Zhu, Yisheng Chen, Zhijie Zhao, Changli Wang, Xiaomin Zhang

**Affiliations:** ^1^ Faculty of Anesthesiology Changhai Hospital Naval Medical University Shanghai China; ^2^ Basic Medical University Naval Medical University Shanghai China; ^3^ School of Anesthesiology Naval Medical University Shanghai China; ^4^ Beijing Institute of Basic Medical Sciences Beijing China; ^5^ Fujian Key Laboratory of Toxicant and Drug Toxicology Medical College Ningde Normal University Ningde China; ^6^ Department of Plastic and Reconstructive Surgery Shanghai Ninth People's Hospital Shanghai JiaoTong University School of Medicine Shanghai China; ^7^ Department of Otolaryngology Naval Medical Center of PLA Navy Medical University Shanghai China

**Keywords:** brain–computer interface, communication barriers, electrode materials, movement disorders, neuropsychiatric disorders, psychiatric disorders

## Abstract

Brain–computer interface (BCI) technology has emerged as a crucial interdisciplinary advancement in the field of neuropsychiatric disease treatment. With the global rise in the prevalence of neurological and psychiatric disorders, which impose a substantial burden on society, BCI offers a novel approach. Since the discovery of bioelectric phenomena in the 19th century, various classification frameworks have been developed based on signal paradigms, invasiveness, and feedback mechanisms. BCI applications span multiple disease areas. In movement disorders, it aids in restoring motor function through prosthetic control, functional electrical stimulation, and brain stimulation–based therapies. For patients with communication barriers, it enables alternative communication methods and speech‐related neural signal decoding. In psychiatric conditions, BCI shows growing potential in both diagnosis and treatment, particularly in conditions like autism and depression. Despite significant progress, BCI faces challenges. The long‐term biocompatibility of electrodes and the resolution of neural signals remain to be improved. To address these limitations, research on new electrode materials, such as carbon nanomaterials and composites, is ongoing. Emerging BCI technologies, including endovascular BCI and optogenetics BCI, present new possibilities. The integration of multimodal technologies and artificial intelligence in BCI systems is expected to enhance performance and enable more personalized treatment. Overall, BCI technology holds great promise for improving the quality of life of patients with neuropsychiatric disorders and driving innovation in the medical and neuroscience fields.

## Graphical abstract

This study investigates the potential of brain–computer interface (BCI) technology in treating neuropsychiatric disorders, such as movement and communication barriers. Our review examines the history, signal paradigms, and diverse applications of BCI while also discussing ongoing research into novel materials and emerging technologies that offer promising future directions. The study provides a comprehensive exploration of BCI applications, deepening the understanding of this innovative technology and its role in shaping the future of medical and neuroscience research by highlighting its potential benefits and challenges.

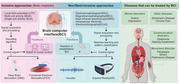

## 1 Introduction

The global incidence of neurological and psychiatric diseases is rising, driven by factors such as population aging, extended expectancy, and psychological stress, imposing significant socioeconomic burdens on families, healthcare systems, and economies. According to 2021 statistics from the Global Health Data Exchange, an estimated 2.87 billion people worldwide are affected by neurological diseases such as age‐related dementias, neurodegenerative diseases, strokes, and traumatic brain injuries, while 1.09 billion suffer from psychiatric conditions, such as depression. Notably, the global prevalence of depression alone reached 320 million cases in 2021, equivalent to 43 cases per 1000 individuals. Parkinson's disease (PD) affects 149 individuals per 100,000 worldwide and has led to the loss of an estimated 7.47 million healthy life years. Globally, the total number of dementia cases attributable to Alzheimer's disease (AD) and other causes approaches 55 million [1]. In the same year, there were about 12 million stroke incidents worldwide, with post‐stroke cognitive impairment observed in 53.4% of survivors [2]. Moreover, these disorders or the underlying neuropathological mechanisms often interact to exacerbate disability [3]. In summary, neurological and psychiatric diseases have become a major global public health crisis demanding urgent development of innovative therapies. However, the adult human brain has limited repair capacity, and neurological damage remains largely irreparable despite decades of intensive research in regenerative therapies.

Brain–computer interface (BCI) technology is a promising research direction for the treatment of neurological and psychiatric diseases. These BCI systems provide users with brain output channels that convert neural activity into control instructions for external devices [4]. Additionally, BCI can provide input signals in the form of motor, tactile, or visual feedback to support rehabilitation training and enable personalized, closed‐loop neuromodulation therapies [5, 6]. The emergence of BCI is redefining the boundaries of modern medicine and providing a new technical path for the treatment of neurological and psychiatric diseases.

A typical BCI system consists of three key components: a signal acquisition module, a signal processing module, and controllable external devices [7]. Currently, the application of BCI in the medical field primarily focuses on neuropsychiatric diseases, including disease assessment, functional assistance, rehabilitation training, and neuromodulation. For diagnostic purposes, BCI systems can detect the onset of neurological impairments or events such as seizures. For functional assistance, BCI‐generated output signals enable patients with strokes, limb defects, or spinal cord injuries (SCIs) to control external devices such as wheelchairs and prosthetics. In the context of rehabilitation, BCI can provide essential feedback and training paradigms. Furthermore, BCI can be utilized to trigger neuromodulatory inputs such as deep brain stimulation (DBS) to suppress seizures or regulate movement in PD [5, 8, 9]. These BCI systems can also be designed for the evaluation and treatment of psychiatric disorders such as depression, autism, attention deficit hyperactivity disorder (ADHD), and bipolar disorder through stimulation‐based or pharmacological interventions [10, 11, 12, 13]. Although the potential of BCI is promising, several challenges remain, including the risks associated with signal detector and stimulator implantation, limited system portability, and the need for improved neural signal decoding for personalized device control. This review summarizes recent advances in the design of BCI systems for neuropsychiatric diseases, including progress in signal detection and decoding, feedback control for external devices and neuromodulation, technical achievements in system engineering and fabrication, and potential future applications.

## 2 History of Brain–Computer Interfaces

The development of BCI systems can be traced back to the discovery of bioelectric phenomena in the 19th century [14]. In 1875, British physicist Richard Caton was the first to record electrical activity from the brains of animals and published his groundbreaking findings in the *British Medical Journal* [15]. This discovery ultimately paved the way for the routine clinical use of electrophysiological measurements such as the electroencephalogram (EEG), electrocardiogram, electromyogram (EMG), and compound nerve action potential recordings for diagnosis, while also laying the foundation for BCI technology development. In 1913, Prawdicz‐Neminski was the first to capture seven distinct patterns of bioelectrical activity in animal brains, terming them components of the “electrocerebrogram.” However, he misinterpreted these signals as artifacts caused by human interference rather than recognizing them as originating from biological processes [16]. In 1924, Hans Berger successfully recorded the first human EEG and identified endogenous oscillating waveforms, which are now recognized as *α*‐ and *β*‐waves [17]. This milestone provided a crucial theoretical foundation for the subsequent development of BCI technology.

The mid‐20th century witnessed significant progress in BCI development. In 1964, Walter et al. first measured slow cortical potentials (SCPs), which were later adapted to enable communication for patients with locked‐in syndrome (LIS)—a condition characterized by full awareness but complete paralysis (also known as pseudocoma) [14, 18]. In 1969, Eberhard Fetz conducted the first BCI experiments with monkeys, marking a pivotal breakthrough. Around the same period, M.B. Sterman identified the sensorimotor rhythm (*μ*‐rhythm) in feline brains [19]. Despite these advances, the term “brain–computer interface” wasn't formally introduced until 1973, when Jacques J. Vidal of UCLA developed a system based on visual event‐related potentials (ERPs), thereby establishing BCI as a distinct research field [20]. A landmark achievement followed in 1978 when William Dobelle implanted a 68‐electrode array in a blind patient's visual cortex. Electrical stimulation successfully induced phosphenes (light perception without actual light input), representing the first major human application of BCI technology [21].

These achievements were followed by important breakthroughs in BCI signal detection and processing technology. In 1988, Farwell and Donchin proposed the famous “P300 speller” paradigm, which allowed individuals with severe motor impairments to communicate by identifying selected letters on a screen through the output of the P300 ERP waveform associated with decision‐making [22]. In the early 1990s, Pfurtscheller and colleagues further expanded the application of BCI by using event‐related desynchronization (ERD) and event‐related synchronization as BCI control signals [23]. In 1992, Erich E. Sutter developed a BCI system based on visual evoked potentials (VEPs) [24], and in 1996, McMillan et al. introduced a system utilizing steady‐state visual evoked potentials (SSVEPs) [25]. Further advancements were made in applying BCI to control motor devices. In 1998, Philip Kennedy and Roy Bakay of Emory University implanted a “motor neuroprosthesis” in a patient with LIS, enabling the control of a computer cursor using BCI [26]. In 1999, the first BCI International Conference was held, marking a key step toward the systematic classification and standardization of BCI technology. In 2000, Jonathan Wolpaw proposed a comprehensive definition of BCI, laying a theoretical foundation for subsequent research [27].

**FIGURE 1:**
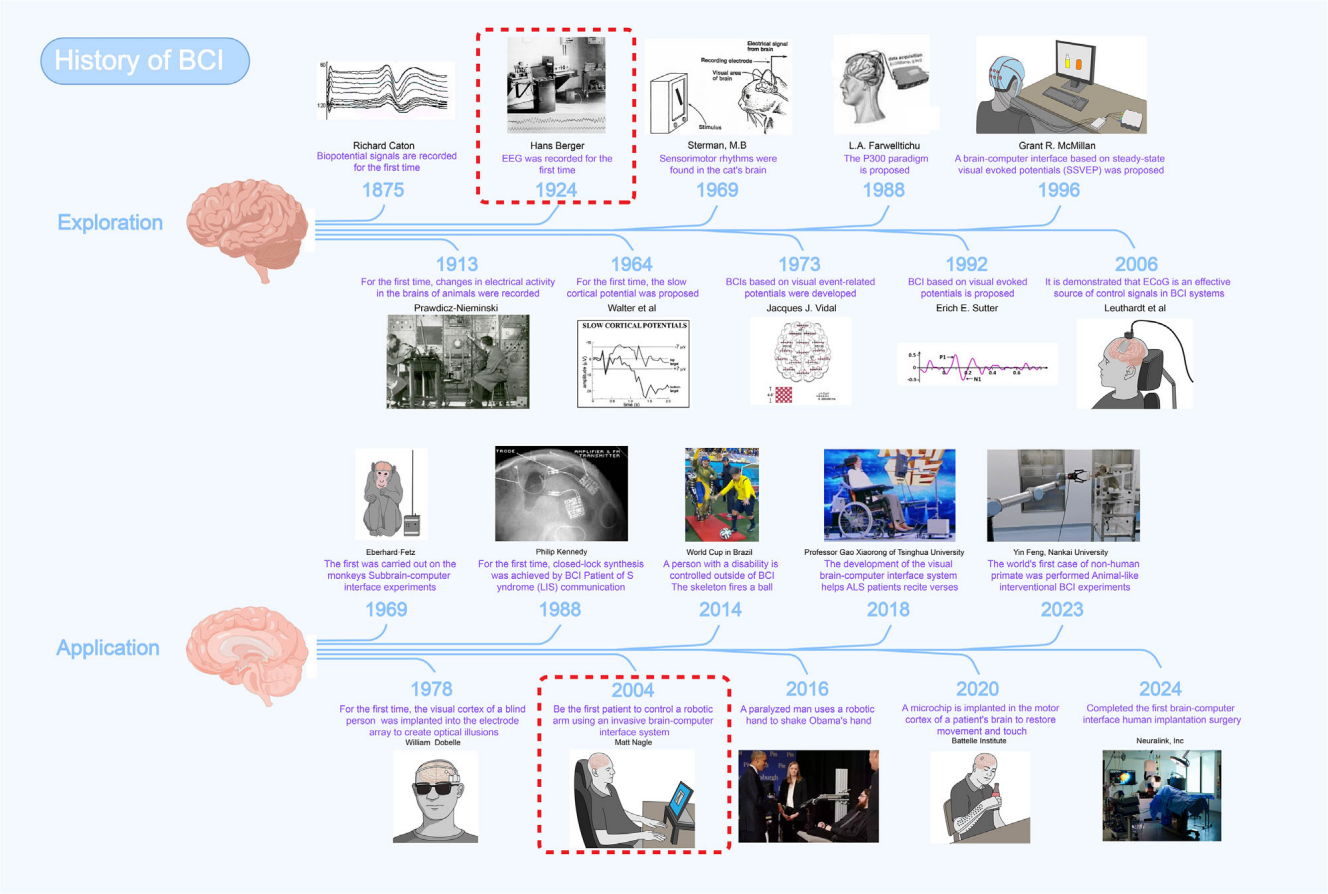
History of brain–computer interface development. Timeline of major technical advances leading to the development of brain–computer interfaces (BCIs). This chart divides the history of BCI development into two eras, exploration and application. During the exploration era, many of the biological signals now used for BCI were discovered, including the electroencephalogram in 1924. During the application era, may BCI systems were developed and first tested on experimental animals. The milestone event in this era was the demonstration that a patient could control a robotic arm using brain signals from implanted electrodes (2004).

The progress in BCI system development accelerated in the early 21st century. In 2004, Matt Nagle was the first patient to use an invasive BCI system to control a robotic arm, marking a major breakthrough in the clinical application of BCI technology [28]. Since then, both experimental and clinical applications of BCI technology have expanded rapidly. Particularly over the past decade, numerous BCI‐based applications for humans have been developed and tested, attracting increasing attention from both the academic community and the news media. Figure 1 presents a timeline illustrating these developments to provide a reference for future research.

## 3 Classification of Brain–Computer Interfaces

The general goal of BCI systems is to enable direct interactions between the human brain and external devices. This can be achieved through converting neural signals into specific output commands (e.g., for motor prosthesis control), converting external stimuli into brain signals for artificial sensory perception, or facilitating reciprocal communication, as seen in sensorimotor feedback systems. These BCI systems can be categorized based on signal measurement modality, application, and invasiveness, among other factors, with each category exhibiting distinct advantages and limitations in signal acquisition, processing, and specific application scenarios. This section systematically describes the classification of BCI systems based on signal source, electrode implantation mode, and feedback mechanism and states their potential applications for neuropsychiatric diseases to provide theoretical support and a technical reference for research and practice in related fields.

According to the signal source, BCIs can be divided into SSVEP‐based, P300‐based, and Melectromyography Imagery (MI)‐based. Steady‐state VEPs are brain waves produced in synchrony with visual stimuli [29]. Through this mechanism, for example, a user can generate control signals at a specific frequency in response to visual targets presented at that frequency. The P300 potential is a transient ERP produced when the brain receives an unexpected stimulus. Finally, MI refers to the electrical signals generated when imagining the preparation or performance of a movement [30, 31, 32]. These signals can be further divided into evoked and spontaneous signals [33, 34]. Evoked activity refers to the neural electrical potentials generated in the brain upon external stimuli, such as SSVEPs and P300 waveforms [35], while spontaneous activity refers to neural activity generated by the user, such as MI [36, 37]. In addition, other BCI signal paradigms are also worth exploring, such as error‐related negativity (ERN), movement‐related cortical potential (MRCP), SCP, and ERD [38, 39, 40]. These paradigms have found particular utility in specific applications. For instance, the ERN reflects a neural mechanism for performance monitoring and error correction, with larger ERN amplitudes being correlated with enhanced error detection and compensatory behaviors [41]. The ERD involves the attenuation of EEG power in the alpha and beta frequency bands prior to movement and is widely applied in motor imagery and execution tasks [42]. The MRCP exhibits distinct spatiotemporal patterns during movement preparation and execution. Studies have combined MRCP and ERD to elucidate the temporal dynamics of motor control [43]. Meanwhile, the SCP has been investigated for its role in SCP modulation, providing a low‐frequency alternative for BCI control [44].

Depending on the location of electrode implantation, BCIs can be divided into invasive, semi‐invasive, and non‐invasive types. Electrodes for noninvasive BCI are placed outside the skin, such as for conventional EEG recording [45, 46] or magnetoencephalography (MEG) [33, 34]. Electroencephalographic electrodes record electrical activity generated by vertical currents on the scalp surface, while MEG measures the magnetic field generated by a tangential current. A semi‐invasive BCI is an intermediate technology between non‐invasive and fully invasive approaches. Its electrodes are implanted inside the skull but on the surface of the cerebral cortex (epidurally or subdurally) without penetrating the brain parenchyma [35]. Electrodes can be surgically placed on the cortical surface (e.g., via electrocorticography [ECoG]) or minimally injected as flexible components (e.g., hydrogel or mesh‐like structures) into target brain regions [47]. This approach combines the advantages of non‐invasive methods (lower surgical risk) and invasive methods (higher signal quality): the absence of parenchymal penetration provides significantly superior spatial resolution compared to electroencephalography (EEG), while posing lower immunological response risks than fully implanted electrodes [48, 49]. In the most invasive configuration, multi‐unit EEG electrode arrays are implanted on or beneath the cortical surface to capture neuronal signals generated near the electrode tips [50, 51]. ECoG electrodes can be placed either on the surface of the cerebral cortex to record synchronized neuronal activity or implanted into gray matter, depending on the application scenario [50, 52]. While non‐invasive BCIs such as EEG‐based systems are the safest, most secure, and least expensive, requiring no surgical implantation, the signal‐to‐noise ratio (SNR) and spatial resolution are comparatively poor, and signals can be easily distorted by external signals and movement. Alternatively, invasive BCI systems achieve the highest SNR but require implantation surgery and may trigger an inflammatory response at the implantation site. Semi‐invasive BCI strikes a balance between the two [53].

**FIGURE 2:**
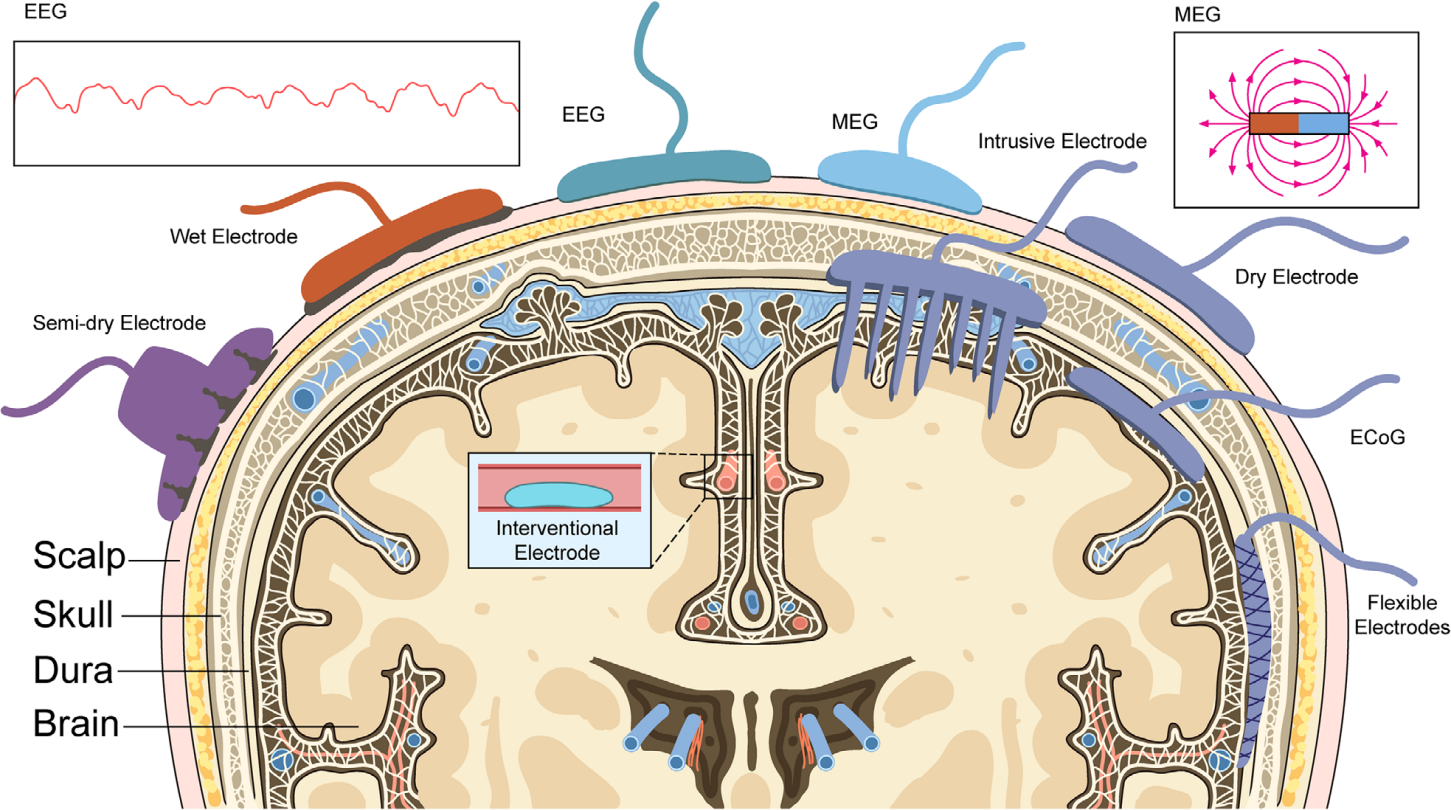
Placement of different electrode types used for BCI signal acquisition and interventional stimulation. Electrodes used for BCI output are classified as invasive, semi‐invasive, and non‐invasive according to location. Non‐invasive electrodes can be divided into dry, semi‐dry, and wet types according to the requirement for conductive paste between the electrode and the skin. Invasive electrodes are located on the cerebral cortex, within brain structures, or in the neurovasculature, while semi‐invasive electrodes are usually located between the skull and the cerebral cortex. Non‐invasive electrodes are located on the scalp surface, such as electroencephalogram (EEG) electrodes and magnetoencephalography (MEG) electrodes. Electrocorticography (ECoG) electrodes can be placed within or outside the cerebral cortex (in the figure, these electrodes do not invade the cerebral cortex). Non‐invasive wet electrodes require a conductive liquid between the electrode surface and scalp, while semi‐dry electrodes contain conductive liquid. Dry electrodes require no conductive liquid between the electrode surface and the scalp. In addition, an interventional electrode is shown, which in this case is placed in an intracranial blood vessel.

Non‐invasive electrodes can be further divided into dry electrodes, wet electrodes, and semi‐dry electrodes depending on whether the application of conductive pastes is required. Dry electrodes do not require conductive pastes, but the higher input impedance at the skin surface results in relatively poor signal quality [54]. In contrast, conductive pastes used for wet electrodes can reduce skin impedance, improve signal quality, and reduce motion artifacts. However, their adhesion may require the removal of underlying hair and cuticle, resulting in skin damage. Moreover, conductive pastes can dry out due to evaporation, resulting in decreased conductivity. Therefore, wet electrodes are not appropriate for long‐term signal recording [7, 55]. Semi‐dry electrodes combine the ease of use of dry electrodes with the higher signal quality of wet electrodes. These are typically made of flexible polymer materials or nano‐metal porous materials and contain electrolytes that are automatically released and penetrate the stratum corneum (outermost epidermal layer) during use, thereby reducing impedance and improving signal quality [56, 57]. These different electrode types and typical locations are illustrated in Figure 2.

BCI systems can be classified as open loop or closed loop based on feedback mechanisms. Open‐loop BCIs analyze brain states in real‐time to control external devices but lack bidirectional signal flow, limiting adaptive adjustment [58, 59, 60]. For example, the P300 speller paradigm [22] enables communication by detecting ERPs in response to visual stimuli, but it does not integrate user feedback to optimize performance. This system is suitable for static tasks like letter selection but may struggle with dynamic adjustments. In contrast, closed‐loop BCIs integrate real‐time feedback to modulate stimulation parameters or decoding strategies. These closed‐loop systems typically utilize neurofeedback (NF) to analyze brain activity and initiate neuroplasticity or modulate brain activity through brain stimulation techniques [61]. For instance, adaptive deep brain stimulation (aDBS) systems [62, 63] dynamically adjust stimulation intensity based on subthalamic nucleus local field potentials (STN‐LFPs) in PD patients, reducing motor fluctuations and improving therapy precision [64]. Another BCI system based on EEG incorporates adaptive closed‐loop control algorithms that automatically adjust feedback patterns and control strategies according to individual differences and training progress, thereby achieving more effective rehabilitation training [65]. Closed‐loop systems demonstrate higher decoding accuracy and personalized intervention [66], though they require more complex hardware and risk feedback‐induced neural adaptation [67].

## 4 Application of Different Brain–Computer Interface Configurations for Disease Treatment

### 4.1 Movement Disorders

Movement disorders are the primary manifestations of many neurological diseases. Patients with PD, for instance, suffer from progressively worsening motor symptoms such as tremors, stiffness, and gait disturbances [68]. While medications can relieve these symptoms in the early stages, they usually lose efficacy with disease progression. On the other hand, BCI systems can be designed to monitor activity in the motor cortex, decode these signals with the assistance of various algorithms (including machine learning), and send decoded commands to control external devices such as robotic arms and stimulators [69, 70]. Since Matt Nagle became the first patient to use an invasive BCI system to control a robotic arm in 2004 [71], the use of BCI to improve movement disorders has shown ever‐increasing promise for the replacement of lost motor function. In recent decades, a wealth of new technologies have been developed that can be applied to the brains or limbs of patients to treat various movement disorders. This section will describe BCI systems for the treatment of movement disorders according to the technologies applied rather than specific diseases, because the treatment methods often overlap.

#### 4.1.1 Prosthetics/Robotic Arms

SCIs can result in life‐altering paraplegia or quadriplegia. For patients with these symptoms, BCI systems using implanted electrodes can record neural activity from the motor cortex and decode this activity into control signals interpretable by prosthetic limbs and other assistive devices. In addition to unidirectional open‐loop systems for external device control, a bidirectional BCI system for an SCI‐induced paraplegia male patient has been described that not only decodes neural activity from the motor cortex to control a robotic arm but also provides tactile feedback by delivering intracortical microstimulation to the sensory cortex for enhanced control [72]. In another application, a subcortical stroke patient implanted with the BCI system was able to decode EEG signals associated with imagined hand movements and control an exoskeleton for opening and closing actions [73]. Further advancements have been made in decoding brain signals to control prosthetic fingers. In one study, researchers recorded and decoded neural activity from the left posterior parietal cortex and MC while patients attempted to move individual fingers and used these signals to control individual prosthetic fingers, offering new possibilities for restoring finer hand function in patients with quadriplegia [74].

To enhance the functionality and control of prosthetics, a hybrid system has been developed that integrates EMG and functional near‐infrared spectroscopy (fNIRS) to control a prosthetic knee through motor imagery (MI) [75]. By integrating these signal detection modalities, this hybrid system effectively mitigates the inherent limitations of each modality (e.g., the low temporal resolution of fNIRS), thereby significantly enhancing the precision and functionality of the prosthetic device. This advancement offers patients a more natural and flexible movement experience.

Additionally, the development of a portable and modular BCI software platform has created new possibilities for home‐based applications [76]. For instance, this platform enables the control of a mechanical glove for grasping as an output device via a smartphone application. This innovation not only simplifies the operation of BCI systems but also offers a more accessible rehabilitation tool for paralyzed patients, significantly improving functional independence.

Several case studies demonstrate the latest advancements in BCI applications for controlling prosthetic devices and robotic arms, particularly in enhancing control accuracy, adaptability, and personalization. A study employed a genetic algorithm (GA) integrated with a support vector machine (SVM) classifier and EEG signals to achieve path planning and target localization for prosthetic devices. This approach generates control signals by simulating the brain activity of subjects imagining arm movements [77]. In another study, fNIRS technology was used to capture brain signals, and signal processing was conducted using the NIRSport 8 × 8 device. Finally, an Arduino board was used to control the prosthetic movement [78]. These examples highlight the potential of BCI in controlling prosthetic and robotic limbs while demonstrating how improvements in system portability, decoding, and feedback control contribute to improved precision and user experience.

#### 4.1.2 Functional Electrical Stimulation

Functional electrical stimulation (FES) of nervous tissue aims to restore function lost due to injury. In most BCI‐FES systems, FES is applied on peripheral nerves via surface (non‐invasive) or implanted (invasive) electrodes to induce muscle contraction and produce movement under the control of brain signals, avoiding spinal cord damage [79, 80, 81]. In addition to movement, FES multi‐stimulation therapy has been used to promote blood circulation and prevent muscle atrophy, significantly improving the daily activities of patients with neurological disorders [82]. By combining BCI with FES, movement deficits due to SCI, PD, or stroke can be improved [37, 83]. Further, these systems can be designed to improve the user experience through feedback during training task performance. For example, Zhang et al. developed an adaptive BCI‐FES system that detects motor intent via EEG and provides visual feedback to dynamically adjust task difficulty based on performance, ultimately improving output control. Subacute stroke patients who received this intervention demonstrated greater improvements in upper extremity motor function, specifically in hand and wrist, as measured by the Fugl–Meyer Assessment for Upper Extremity and these improvements were sustained at six months post‐intervention, suggesting durable recovery of motor function [84].

Despite these successes, significant challenges remain in combining FES with EEG‐based BCI. For instance, while EEG signal analysis (particularly of *μ*‐ and *β*‐waves) can encode MI, it still lacks the precision to identify the intended target muscles [37, 85, 86], hindering accurate modulation of FES parameters such as pulse amplitude and width for the desired output. Therefore, the acquisition of surface electromyography (sEMG) signals is particularly important. The addition of sEMG solves the problem of ambiguous muscle activation intention for more precise FES control [87]. Further closed‐loop BCI control can be achieved by integrating EEG and sEMG signals, where EEG signals are used to detect exercise preparation or execution, and sEMG signals are used to monitor muscle activity and provide feedback for adjusting FES parameters. Based on the combined analysis of EEG and sEMG signals, the BCI system can modulate FES parameters such as pulse amplitude, width, and frequency [88, 89]. With sEMG, BCI‐FES can also assess muscle activity during exercise or rehabilitation, delivering FES to inactive muscles to prevent atrophy [86].

Cross‐talk in hybrid BCI systems, such as electromyography (EMG) artifacts in EEG signals, is effectively mitigated through advanced preprocessing and signal fusion techniques. Independent component analysis (ICA) [90] and wavelet transform [91] are widely employed to isolate muscle artifact components, while spatial filtering methods, such as the common average reference, reduce environmental noise contamination. For signal fusion, canonical correlation analysis (CCA) [92] has demonstrated superior performance in identifying shared features between modalities, particularly in removing EMG interference from EEG signals compared to ICA, due to its robustness in handling non‐stationary and non‐linear artifacts. Deep neural networks, including multi‐stream convolutional neural networks (CNNs) [93, 94], are increasingly adopted to process modality‐specific features separately before integration, enabling end‐to‐end learning of complex patterns. Additionally, hybrid methods such as wavelet transform—ICA [91] and empirical mode decomposition–ICA [95] have been validated for their effectiveness in removing artifacts in noisy clinical environments, especially when combined with real‐time processing frameworks like Gaussian mixture models [92]. These approaches collectively enhance the robustness and accuracy of hybrid BCI systems in challenging operational conditions.

#### 4.1.3 Brain Stimulation

Combining BCI with brain stimulation can be used to improve motor control in PD [51, 96, 97]. Stimulation devices may be either surface‐based non‐invasive brain stimulation (NIBS) or implanted for DBS [98]. Due to safety and ease of application, NIBS modalities are more widely used for the functional rehabilitation of stroke patients [99], including transcranial magnetic stimulation (TMS) and transcranial electrical stimulation (TES) [100, 101]. TES modalities include transcranial direct current stimulation (tDCS) and transcranial alternating current stimulation (tACS), and both have been shown to effectively activate motor neurons in the diseased or non‐diseased hemisphere to drive motor activity [102]. Modulation of the theta‐*γ* band, in particular using tACS, has been shown to significantly improve motor skills acquisition [103]. DBS targeting the basal ganglia (especially the STN and globus pallidus [Gpi] interna) is now a routine intervention for PD, and several groups have applied BCI‐DBS to treat movement disorders caused by PD and stroke [51, 104, 105]. Others have used cerebellar DBS to enhance motor function recovery in chronic stroke patients and found neuroplastic reorganization of the ipsilateral cortex even years later [106]. A multicenter randomized controlled trial (RCT) is currently underway to evaluate the efficacy of DBS in improving motor dysfunction after stroke. Research has demonstrated the potential of DBS in treating post‐stroke motor dysfunction, such as the use of GPi‐DBS combined with ventral intermediate/ventral oral posterior nucleus–DBS for unilateral post‐stroke dyskinesia and Zona incerta–DBS for patients with post‐stroke hemorrhage [107]. In addition, researchers have found that aDBS based on beta burst can improve gait disturbances and freezing of gait in patients with PD, with efficacy comparable to that of traditional continuous DBS. This technique enhances the precision and effectiveness of treatment by real‐time monitoring and adjustment of stimulation parameters [108]. DBS is effective in treating movement disorders, with specific effects observed in different brain regions. Stimulation of the STNand the internal GPi can improve motor symptoms in patients with PD, such as tremors, rigidity, and bradykinesia [109]. Additionally, STN stimulation has been found to alleviate choreiform symptoms in patients with Huntington's disease, and the GPi is also considered a potential therapeutic target for this condition. For AD, targeting the amygdala and the anterior cingulate cortex can enhance cognitive function. Furthermore, cortical DBS is emerging as a potential therapeutic target for patients with amyotrophic lateral sclerosis (ALS) [110].

Optimization of DBS for BCI applications is also ongoing. Conventional DBS devices produce artifacts during stimulation that interfere with the recording of neural signals. In response, a closed‐loop Alpha DBS system has been developed that effectively removes artifacts through advanced signal processing technology, thereby improving signal quality and allowing the adjustment of stimulation parameters in real‐time according to neurophysiological indicators to achieve more efficient and reliable stimulation effects [62].

#### 4.1.4 Applications of Other Technologies

A BCI system combined with sensorimotor rhythm NF training has been shown to alleviate tetrahydropyridine‐induced Parkinsonian symptoms in non‐human primates treated with levo‐dihydroxyphenylalanine, a ubiquitous drug treatment for early‐stage PD [52]. Briefly, EEG signals are converted into visual or auditory feedback, with the brainwave pattern influencing how feedback is presented [111]. Through practice, reinforcement, and feedback, subjects can learn to control neural activity autonomously [112]. For example, DyNeuMo‐2 NF is a beta oscillatory NF training game for patients with PD, in which users control a game cursor by self‐adjusting brain waves. The user moves the cursor to a specified position by increasing motor cortex *β*‐band power, and improvement through training ultimately enhances exercise capacity [98, 113].

BCI can also be used in conjunction with action observation therapy (AOT), a neurorehabilitation method for patients with movement disorders that utilizes the automatic activation of neural structures responsible for performing an action when observing another person performing that action (mirror‐neuron effect) [114]. Indeed, several studies have used AOT to treat patients with chronic ischemic stroke (defined as still recovering more than 6 months after the event), as well as PD, cerebral palsy, aphasia, and multiple sclerosis [115, 116, 117, 118]. Functional magnetic resonance imaging (fMRI) has revealed a marked increase in mirrored brain region activity in patients treated with AOT [119, 120]. Other research groups have combined AOT, sEMG, and FES (AOT‐BCI‐sEMG‐FES) for rehabilitation. In this scenario, the patient wears a helmet‐mounted EEG sensor and sEMG sensor while receiving AOT treatment. In addition, an inertial measurement unit is used for motion tracking to assess whether the patient has completed the maneuver correctly, while FES is adjusted based on this feedback to assist in completing the maneuver [86]. This system may apply to a broad spectrum of neurological and musculoskeletal diseases for rehabilitation and exercise training, among other interventions.

In addition, game technology is being used to treat patients with quadriplegia. Willsey and colleagues have developed a new high‐performance intracortical brain–computer interface (iBCI) system that can decode the brain activity of paralyzed patients and control the movement of virtual fingers. In the application study, a 69‐year‐old male with quadriplegia due to SCI was implanted with two 96‐channel silicon microelectrode arrays (MEAs) into the “hand tubercle” area of the left anterior central gyrus to record brain activity. In the training phase, the patient played games such as navigating virtual obstacle courses and random circle crossings with virtual quadcopters by controlling the movement of virtual fingers through brain activity. The results suggested that this iBCI system can achieve highly refined finger control and provide a new way of entertainment and socialization for paralyzed patients [121].

To date, the application of BCI in the management of motor disorders has demonstrated considerable breadth, with extensive experimental applications observed in conditions such as PD, epilepsy, and stroke. Through the utilization of advanced technologies, including robotic arms, FES, and DBS, BCIs have successfully enabled bidirectional intervention between brain regions and limbs, thereby improving patients’ motor functionalities. The incorporation of virtual reality (VR), gaming technologies, behavioral observation therapy, and NF has further augmented the therapeutic efficacy for individuals with motor disorders. Ongoing advancements aimed at enhancing the sensitivity and signal precision of existing BCI systems are continuously being pursued, thereby facilitating performance optimization and broader applications of BCI technology. Nevertheless, despite the progressive achievements in relevant therapeutic domains, the majority of explorations remain confined to the experimental realm, clinical translation, and practical implementation, posing substantial challenges within the current research landscape. The invasive risks and potential complications associated with BCI interventions cannot be overlooked. Additionally, the portability of BCI treatment modalities should be considered to enhance patient convenience and alleviate treatment burdens. Future BCI therapeutic paradigms should aspire toward a personalized approach, targeting specific brain regions and muscles to refine treatment precision, enhance therapeutic outcomes, and minimize unnecessary harm.

### 4.2 Communication Barriers

In addition to movement disorders, communication disorders are also common symptoms of neuropsychiatric diseases such as ALS and LIS and are prevalent among individuals with hearing loss and injuries affecting cortical speech production (dysphonia). In ALS, motor neurons in the brain and spinal cord degenerate, resulting in motor‐related communication impairments [50], while LIS is a pervasive disorder of consciousness affecting the ventral and caudal sides of the pontine and midbrain [122]. Some patients with LIS have partially preserved motor ability and can communicate through vertical eye movements and eyelid movements, but those with complete LIS are unable to blink or gaze directionally to communicate [123, 124]. Disorders similar to LIS, including covert consciousness, cognitive‐motor dissociation, minimally conscious state (MCS), and higher‐order cortical motor dissociation, also feature profound communication deficits [125]. In addition, congenital deafness, auditory nerve damage, and otosclerosis substantially limit communication. Here, we describe BCI technologies that provide several strategies to solve communication barriers, whether due to vocal or auditory impairments.

#### 4.2.1 Dysphonia

Patients with ALS inevitably lose verbal communication capacity in the later stages of the disease, but various BCI configurations can be applied to replace voice communication. One group implanted a high‐density multi‐electrode array on the surface of the cortical speech control area in people with aphasia, and they also applied deep learning algorithms to create computational models for decoding the measured signals into the intended words and sentences. The system demonstrated an average decoding speed of 15.2 words per minute, accompanied by an average word error rate of 25.6% [126]. In addition, a BCI coupled with ECoG, linear judgment analysis, and CNNs was used to decode speech‐related neural signals representing different levels of speech, such as phonemes, words, and sentences. In addition, the BCI system was followed by the use of vocoders combined with deep learning algorithms (regression probability models and phase estimation models) for speech synthesis [122, 127]. A core advantage of this method is greater communication speed compared to traditional eye‐tracking methods, in which gaze direction is used to indicate the selection of individual letters or words from an array. In one such study, researchers implanted four MEAs into the brain of a patient with ALS to record neural activity during attempted oral movements, pronunciation, and speech. And they also used recurrent neural networks to decode this neural activity and convert it into text. Compared to earlier speech interface systems and eye‐tracking technologies, this system achieved an even higher decoding rate (62 words/min) and a lower word error rate (9.1%) [128].

Some of the BCI techniques described for movement disorders can also be used by LIS patients to facilitate indirect communication. For example, BCI plus FES can be used to decode individual gestures, American Sign Language logos, and handwritten characters based on neural activity, producing output signals to control FES for improved sign language and handwriting. Alternatively, this system can decode information about facial activity and facial expressions from neural activity and then induce facial movements to form appropriate facial expressions [79].

In summary, most current BCI systems applied to vocal disorders combine classical learning algorithms, deep learning algorithms, and data models to translate neural signals into words. Compared to traditional methods like eye tracking during letter selection, these systems are faster and more accurate. With an increase in computational power and the accumulation of neurophysiological data relating EEG signals to language content, the scope of these applications will certainly expand. For example, by incorporating artificial intelligence (AI) technology, BCI systems could one day be able to analyze brain signals in real‐time and dynamically adjust the decoding strategy through closed‐loop stimulation. Such a real‐time feedback mechanism would not only improve the response speed of the system but also optimize the stimulation parameters because of the immediate need for further improvement of the treatment effect. With the help of AI algorithms, BCI systems could also create personalized decoding models based on the patient's individual EEG activity patterns, linguistic abilities, communication styles, and clinical symptoms. A BCI big data platform based on cloud computing could also be built to process, analyze, and share EEG data for collaborative research. This kind of platform would not only provide rich data support for the optimization of BCI algorithms but also predict the user's intent through machine learning, further improving decoding efficiency and accuracy. In addition, data sharing can help reduce research costs and promote improvements and innovations in BCI technology. In short, future applications of BCI technology for the treatment of dysphonia could be more intelligent, personalized, and applicable. By combining AI technology, big data analysis, and closed‐loop control strategies, BCI systems are expected to achieve breakthroughs in decoding accuracy, treatment efficiency, and user experience, allowing more efficient and accurate communication.

#### 4.2.2 Hearing Impairment

Currently, the primary modality used for hearing impairment therapy is direct current stimulation (DCS). In recent years, DCS has been used to induce a variety of perceptions in humans, including sensorimotor, auditory, visual, and phonological. Hearing and other complex perceptions can be evoked by directly stimulating various regions of the primary auditory cortex, including Heschl's gyrus and the temporal lobe, particularly the superior temporal gyrus, where stimulation can cause auditory hallucinations (dripping, buzzing, and vocals) [129]. Hong and colleagues have developed a closed‐loop speech BCI brain stimulation system with a decoder that converts silent speech into signals that, in turn, are used to synthesize audible speech. AI‐based decoders are also used to analyze the speech of others, extract relevant language or sound features, map them to specific brain regions, and then induce corresponding speech perceptions in the user [130]. While there has been some progress in the application of DCS to treat auditory impairments, there are several outstanding challenges to evoking the perception of artificial speech. First, the brain regions involved in speech cognition are highly distributed and complex at both the regional and network levels; neural activity in speech areas is often reduced under electrical stimulation, and it is difficult to induce recognizable speech perceptions. These issues have resulted in inconsistent perceptions induced by stimulation of the same area across patients [130, 131, 132]. In response to these problems, some research groups have instead included the use of visual speech to enhance the recognition and processing of auditory speech. A cross‐modal interaction between the visual and auditory cortices has been found [133], suggesting that the use of both visual and auditory speech may be more effective. In addition, multi‐site stimulation has shown promise for improving the efficacy of speech coding. For instance, a multi‐point stimulation protocol was used to improve DCS‐evoked speech perception by first identifying nodes that display activity during a working memory task and then simultaneously stimulating both nodes to achieve improved performance [134].

For individuals with hearing impairments, speech communication in multi‐speaker environments poses significant challenges. While people with normal hearing can effortlessly shift their attention from one speaker to another, this task is considerably more difficult for those with hearing disabilities [135]. However, BCI technology combining advanced algorithms may assist hearing‐impaired individuals in focusing on specific conversations in noisy environments. To this end, several groups have developed brain‐controlled hearing assistance systems that integrate auditory attention decoding with either binaural speech separation models [136] or single‐channel speech separation algorithms [137]. By analyzing intracranial electroencepholography or invasive ECoG data, these systems can separate multiple speech streams while preserving spatial information. Besides, they can also predict motion trajectories to enable the effective identification and enhancement of the speaker of interest while maintaining the spatial context and speech quality of the conversation [136, 137]. This model incorporates multiple speakers, natural background noises, and turn‐taking conversations, with speakers moving dynamically in space, making it more realistic and applicable to real‐world scenarios. Such advances hold significant potential for broader application, offering hearing‐impaired individuals a more natural and effective way to engage in multi‐speaker communication.

However, research on BCI therapy for hearing impairment is still in the preliminary exploration stage, and the number of related studies is limited, with most still at the laboratory research level. Unlike the BCI model for movement disorders (signaling from the central nervous system to peripheral effectors and, in some cases, relatively primitive feedback), the BCI treatment model for hearing impairment requires the transmission of complex sensory information from the peripheral auditory system to the central auditory cortex for precisely patterned activation of cortical circuits. However, the individual structures and network organization of the primary auditory cortex, secondary auditory cortex, and higher auditory sympathetic cortex are highly complex, and precise localization and stimulation still present great challenges. Thus, existing BCI technologies can only produce simple auditory perceptions, such as single‐frequency pure tones, which are insufficient for speech recognition and complex sound processing. In addition, the auditory perception generated by BCI stimulation is random and unstable, and it is difficult to generate the same cortical speech response repeatedly to the same input, so these systems are not ready for actual communication scenarios.

**FIGURE 3:**
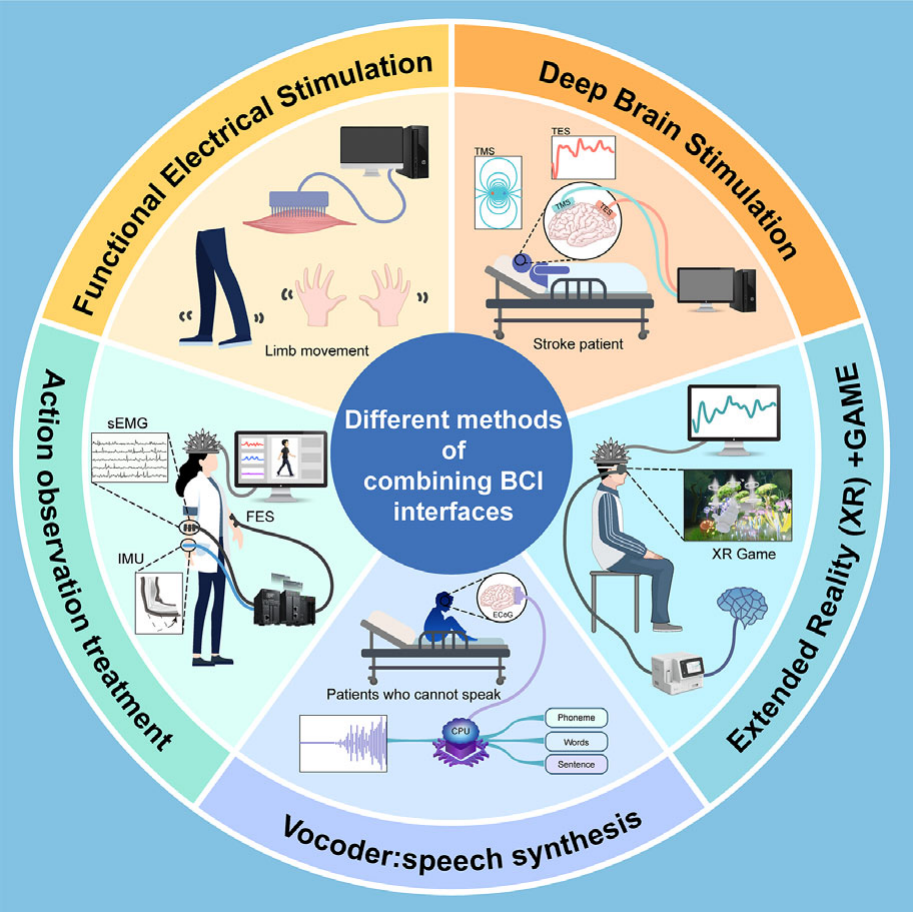
BCI combined with other technologies. BCI can be used in combination with a myriad of other technologies, including functional electrical stimulation (FES), deep brain stimulation (DBS), extended reality (XR) + gaming, vocoder, and action observation therapy (AOT), to diagnose and treat diseases. The FES component directly stimulates muscles (e.g., for limb movement) via electrodes controlled by system output. The DBS component shown here has two electrodes, one for transcranial magnetic stimulation (TMS) and the other for transcranial electrical stimulation (TES). Electrodes for DBS may also be inserted within specific brain structures, such as the basal ganglia for Parkinson's disease treatment. In the XR + gaming panel, the user wears virtual reality (VR) glasses to play a game, and the signal collection and processing modules provide feedback stimulation to the brain for improved performance (or external device control). The vocoder component collects the EEG signal of aphasia patients through an ECoG electrode, analyzes the phonemes, words, and sentences, and then synthesizes sound waves to generate artificial speech. The AOT panel shows the user observing the movement of others on the display, which activates mirror neurons in the brain controlling the same action. Surface electromyography (sEMG) is used to measure muscle movement, and an inertial measurement unit (IMU) is used to measure body movement trajectory. FES uses information from sEMG and IMU to control the muscle activation pattern.

Nevertheless, by exploring the neural mechanisms of speech perception and processing in detail and clarifying the functional characteristics of the auditory cortex and its related neural networks, we can provide a theoretical basis for precise stimulation and the induction of cortical responses perceptible as speech. It is expected that the complexity and stability of auditory perception will be improved by developing synergistic stimulation schemes for multiple auditory‐related brain regions and by combining multiple parameters (e.g., frequency, intensity, timing) to optimize the stimulation pattern. In addition, a closed‐loop BCI system based on real‐time NF has been developed that can dynamically adjust stimulation parameters according to the patient's neural activity for adaptive regulation, thereby improving the treatment effect. The combination of AI and deep learning technologies to optimize the processing and decoding of EEG signals will also further improve the accuracy and reliability of BCI systems for auditory perception. The applications of BCI systems with various signal measurement, encoding, and control technologies are illustrated in Figure 3.

### 4.3 Mental Disorders

In recent years, numerous BCI technologies have been investigated for the treatment of mental illnesses. As an emerging cognitive training tool, BCI has shown great potential. Most of these BCI studies have focused on the diagnosis and treatment of depression and autism.

#### 4.3.1 Autism

People with autism spectrum disorder (ASD) often have difficulty sharing attention on a particular object with others, a condition termed impaired joint attention. Common examples of joint attention include following another person's gaze or making a pointing gesture toward an object to establish shared attention. The integration of P300‐based BCIs with VR technology can effectively guide attention toward specific objects [138]. One study reported that P300‐based BCI combined with VR joint attention training on 15 patients with ASD resulted in a higher average accuracy than traditional machine learning methods [139]. This study provides a valuable resource for the further development of P300‐based BCI algorithms for ASD.

People with ASD also have social and cognitive impairments that are strongly associated with deficits in the patient's error‐monitoring process [140]. Pires et al. developed a BCI system based on involuntary NF for cognitive training to help people with ASD improve their error‐monitoring ability [141]. In the application study, 10 healthy subjects and 1 high‐functioning autistic subject were asked to observe the facial expressions (happy or sad) on the screen and judge whether the virtual image's movements were correct based on the facial expression. At the same time, a reinforcement learning algorithm was used to determine the optimal strategy according to subject judgments. In addition, with the help of an error‐related potential (ErrP) detector [142], the EEG signals of the subjects were analyzed in real‐time to identify the neural activity associated with incorrect judgment. The average accuracy of ErrP detection was 77.1% in mode 1 with no feedback (i.e., only observing the agent's learning process) and 81.6% in mode 2 with feedback (showing the agent's learning progress through a green progress bar). All subjects successfully helped the agent in learning the optimal strategy in at least one test, validating the feasibility of the proposed BCI approach and providing a foundation for future clinical trials in the ASD population. This method is expected to serve as an effective tool for cognitive training in ASD populations, helping individuals improve their error detection and social interaction skills.

It is also important to assess and regulate the psychological state of people with autism. In one study, the long short‐term memory recurrent neural network (LSTM RNN) model with BCI‐EEG was applied to identify the psychological stress states of adolescents with ASD and healthy adolescents, and the system achieved an overall accuracy of 93.27%. These findings support the accuracy and generalizability of the two‐layer LSTM RNN model for real‐time assessment and alleviation of psychological stress using EEG‐based BCIs [13]. Another clinical study improved the psychological state of an autistic male adolescent with comorbid ADHD using BCI for entertainment. Further, the patient demonstrated increased interest and participation in related activities, and the effect was superior to other forms of recreation [10].

At present, the application of BCI technology is primarily focused on the treatment of core ASD symptoms such as joint attention disorder, social deficits, cognitive impairments, and negative psychological states. Using these BCI systems, patients receive specialized training on attention, mental state control, cognitive function, and social interaction, thereby improving social adaptability and quality of life. However, current applications of BCI for autism treatment have not yet included direct brain interventions due to the distributed and still largely mysterious nature of the underlying neuropathology. Indeed, autism is a highly heterogeneous neurodevelopmental disorder involving abnormalities in multiple brain regions and neural circuits, making direct neurological intervention especially difficult. Therefore, future research should focus on identifying autism‐related EEG/ERP potentials or other features of neural activity to pinpoint abnormal areas in the brain. It may then be possible to attempt such targeted intervention by combining BCI with DBS, TMS, or tDCS. In addition, future research must explore the combined application of closed‐loop stimulation, NF training, and behavioral observation therapy to improve the therapeutic effect of BCI. For example, a closed‐loop BCI system that automatically adjusts stimulation parameters based on a patient's real‐time brain activity could enable personalized treatment despite the heterogeneity of the disorder. Also, BCI systems combined with AI algorithms are expected to further improve the accuracy of signal decoding, providing more accurate and efficient treatment options for patients with autism.

In conclusion, although the application of BCI technologies for autism treatment is still in the exploratory stage, these systems have shown significant potential to improve the core symptoms. In the future, through in‐depth research on the neurobiological mechanisms of autism, combined with multimodal neuromodulation technology and intelligent algorithms, BCI is expected to become an important treatment tool, bringing new hope to patients and their families.

#### 4.3.2 Depression and Anxiety

Major depressive disorder (MDD) is among the most common psychiatric disorders, but clinical diagnosis can be time‐consuming and ultimately inaccurate [143]. Peelle et al. have developed an automated BCI‐EEG system to classify and score patients with depression based on the spatial distribution of specific EEG frequency bands to improve the success rate of clinical diagnosis [144]. The investigators used two residual neural networks for EEG monitoring during specific tasks to classify depression and score depression severity [145]. A comparison of 52 healthy college students and 48 patients with MDD revealed significantly lower response accuracies and response times on working memory tasks among patients with mild depression compared to controls. This system provides a new method for the early detection and evaluation of depression. Future research is warranted to further optimize the model and explore more effective treatments for depression based on BCI.

In addition, Pizzagalli et al. integrated psychoneurotherapy (PNT) and BCI‐EEG to treat patients with MDD [146]. Analysis of recorded EEG activity revealed excessively strong high‐*β* activity (18–30 Hz), a common abnormality in patients with MDD [147]. The intensity of high‐*β* activity was converted into a digital signal, filtered, and feature extracted so that the BCI system could provide feedback. The intensity of high‐*β* activity was subsequently presented to the patient in the form of a graph, and when intensity exceeded a preset threshold, changes in the graph provided a cue for the patient to regulate their thoughts and emotions. Before and after treatment, patients were assessed for depressive symptoms using a self‐report questionnaire, and it was found that this combined BCI‐EEG plus PNT intervention modulated brain activity and alleviated depressive symptoms. In addition, normalization of high‐*β* activity in the cortical‐limbal/paralimbic region was associated with improvement in depressive symptoms [148].

In another study, 15 patients with anxiety and depressive spectrum disorders were randomly assigned to an experimental and a control group. The experimental group used a Neuro‐Upper device, which analyzes participants' brain responses to auditory stimuli and provides real‐time feedback in the form of flashing lights that match the dominant brain rhythm, while the control group received simple psychoeducation and non‐interactive video viewing [149]. Following the intervention, Hamilton Depression Rating Scale (HAM‐D) scores were significantly lower, and cognition significantly improved in the experimental group compared to the control group. Further, these changes in HAM‐D scores appear to be associated with changes in beta‐1, beta‐2, and delta‐band power, while improvements in cognitive function were associated with changes in the theta band. These findings support the potential of this BCI technique for regulating emotional states and improving cognitive function.

Brain stimulation technology is rapidly gaining acceptance as an alternative treatment for MDD. A systematic review and network meta‐analysis of studies on the application of various non‐surgical brain stimulation techniques for MDD treatment concluded that electroconvulsive therapy (ECT), repetitive transcranial magnetic stimulation (rTMS), theta burst stimulation (TBS), and tDCS can significantly improve the treatment response rate compared to the sham control group. Among these stimulation‐based interventions, bitemporal ECT and high‐dose right unilateral ECT were found to be superior to other stimulation techniques [150]. In addition, DBS has demonstrated efficacy for treatment‐resistant depression (TRD). Several research teams have conducted clinical trials of DBS targeting different brain regions for the treatment of TRD, including the ventral capsule/ventral striatum [151], cingulate ileus subgenicular sulcus [152], and the dorsolateral medial anterior tract [153]. Despite the success of early clinical trials, the use of DBS for TRD treatment still faces many challenges, such as a strong placebo effect, clinical trial design flaws, absence of long‐term follow‐up, heterogeneity of symptoms, and low commercial viability [154, 155]. To address these challenges, researchers are working to develop closed‐loop BCI‐DBS systems able to automatically adjust the stimulation strategy based on the patient's individual disease phenotype and physiological response, thereby improving treatment precision. By identifying specific clinical phenotypes associated with the DBS response, combined with studies of DBS impact on brain activity, physiological indicators of treatment efficacy can be identified. These next‐generation BCI‐DBS systems should also provide response feedback for closed‐loop control. In addition, the introduction of new technologies such as multi‐site stimulation, transcranial ultrasound stimulation, and timing interference stimulation is expected to further improve the efficacy of DBS for depression [137]. Despite the limitations of early clinical trial results, DBS still has significant potential for the treatment of TRD. Through technological improvements, optimization of clinical trial designs, and adjustment of treatment strategies, closed‐loop BCI‐DBS is expected to become an effective means of treating TRD.

**FIGURE 4:**
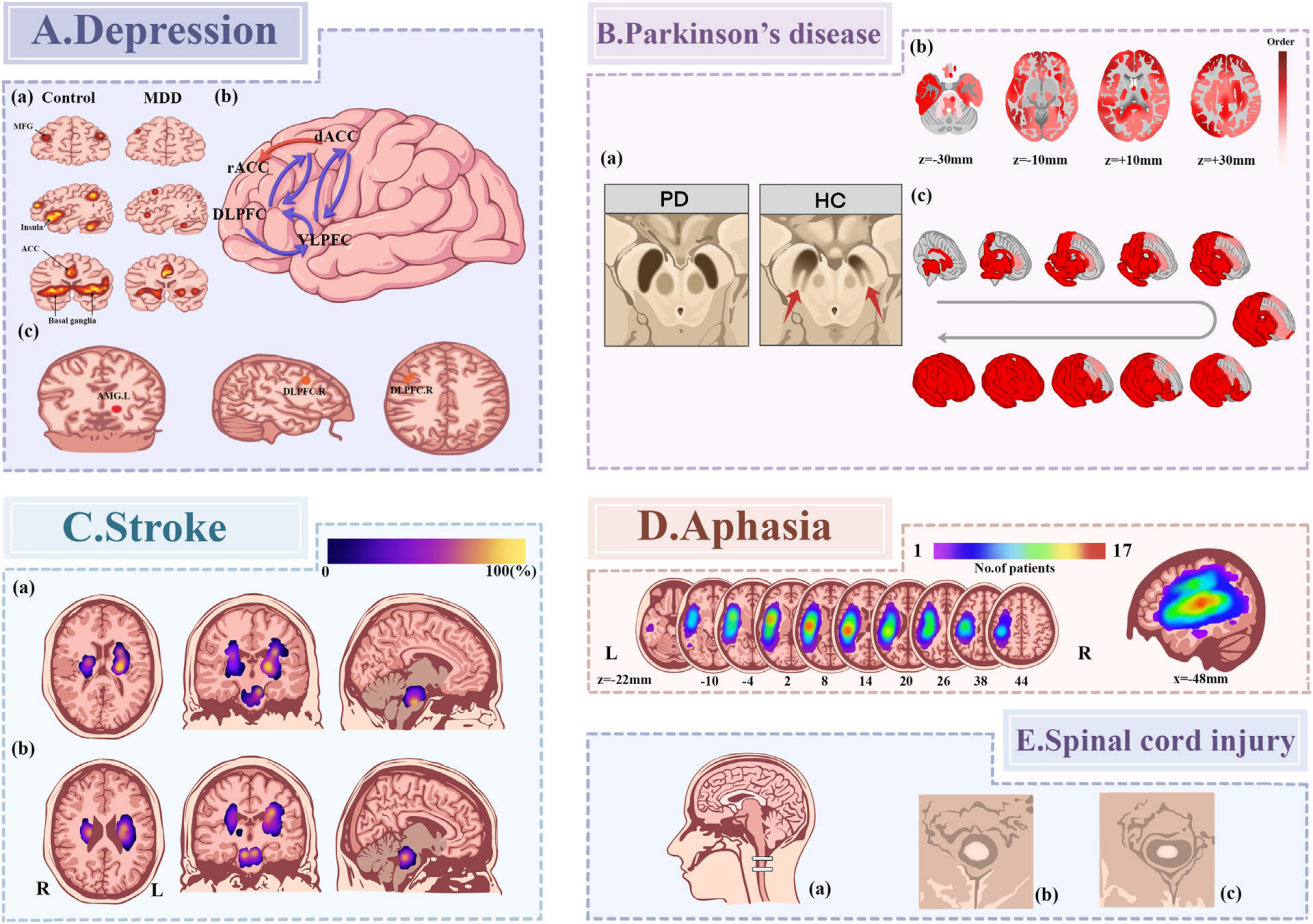
The diseases treatable with brain–computer interface technology. Region A displays a 3D brain model of functional connectivity abnormalities in depressed patients, with markedly reduced connectivity in the anterior cingulate cortex (ACC) (a). Major depressive disorder (MDD) patients exhibit lower activation in the prefrontal cortex (e.g., DLPFC, VLPFC), insula, and basal ganglia (b), and significantly decreased activity in the right DLPFC and left VLPFC under positive emotional interference (c). Region B shows the substantia nigra region in the midbrain. Panel (a) shows the midbrain substantia nigra region. Panel (a) reveals the absence of the high signal (“swallowtail” sign, white arrow) in the dorsal lateral substantia nigra of Parkinson's disease (PD) patients compared to healthy controls (HC), indicating neuronal cell damage in the substantia nigra pars compacta. Panel (b) illustrates the progression of structural changes in PD patients’ brains from early to late stages (white to deep red), while panel (c) shows a snapshot of these changes in a 3D‐rendered brain. White matter (WM) integrity declines first in commissural tracts (e.g., corpus callosum), spreading via association tracts (e.g., cingulum, fronto‐occipital fasciculus) to projection tracts (e.g., corticospinal tract). Cortical gray matter (GM) thinning begins in the somatosensory cortex and spreads through association cortices (parietal, temporal, occipital lobes) to the prefrontal cortex. Region C depicts overlapping lesion areas in stroke patients. “L” and “R” denote left and right hemispheres, respectively. Panels (a) and (b) represent two datasets. The primary lesion sites in stroke patients are concentrated in the basal ganglia and pons. Region D shows overlapping lesion areas in aphasia patients. Color intensity indicates the number of patients with lesions in specific voxels. Numbers below axial and sagittal views denote MNI z‐ and x‐plane coordinates. “L” and “R” represent left and right hemispheres. Lesions are primarily localized to frontal and temporal lobes, suggesting severe involvement in aphasia patients. Region E compares spinal cord cross‐sectional areas between spinal cord injury (SCI) patients and controls. SCI patients exhibit significantly reduced spinal cord cross‐sectional area compared to controls.

The potential application of BCI for the diagnosis and treatment of depression has been studied extensively, including different stimulation sites and patterns. It is hoped that by combining NF training and real‐time feedback mechanisms, BCI technology can help patients learn to regulate neural circuits related to emotion, thereby improving their emotional state and alleviating depressive symptoms. It is already well known that brain stimulation technology can effectively improve the functional activity of depression‐related brain regions (e.g., prefrontal cortex, cingulate gyrus), thereby reducing depressive symptoms and improving treatment effects, so future challenges involve developing BCI‐based control systems for optimal DBS. Treatment may also be improved by combining BCI with emerging technologies such as extended reality (XR), gamified design, and NF. VR‐ or augmented reality (AR)‐based games and appropriate feedback could provide patients with an immersive treatment environment that enhances engagement and adherence. In addition to core symptom relief, these innovative approaches could help improve cognitive functions and social interactions. By further optimizing algorithms, improving signal decoding accuracy, and developing personalized treatment plans, BCI is expected to become an important tool for precision depression treatment. At the same time, BCI systems combining multimodal neuromodulation technology and AI algorithms are expected to achieve more efficient brain region intervention and symptom relief and provide more comprehensive and personalized treatment options for patients with depression. The scope of diseases that may benefit from BCI‐based treatments is illustrated in Figure 4.

### 4.4 Other Diseases

Emerging interventions such as XR and gaming have been shown to improve physiological and cognitive function. Based on these results, several research groups have explored invasive and NIBS strategies in combination with cross‐platform game engines and development platforms such as Unity to create bidirectional XR‐based BCIs [98] to treat disease. For instance, on‐demand DBS targeting the medial temporal lobe combined with XR has been used to enhance memory in AD [108]. Game‐based interventions combined with tDCS have also shown potential for promoting long‐term learning and knowledge retention, including tDCS targeting the dorsolateral prefrontal cortex to improve performance in video games requiring training, learning, and physical movement [156].

Chronic pain is among the most debilitating conditions in terms of its impact on quality of life, and pharmacological treatments often diminish in efficacy over time or cause severe adverse effects, including the risk of addiction. As an alternative, BCI has the potential to improve chronic pain management by detecting pain signals in real‐time and precisely adjusting treatment. Sun et al. reported good analgesic effects using a multi‐region closed‐loop BCI that records LFPs in the somatosensory cortex and anterior cingulate cortex and uses optogenetics [7] or DBS to activate the prefrontal cortex [157].

Several studies have also examined potential BCI‐based treatments for ADHD, post‐traumatic stress disorder, schizophrenia, and visual impairment. Due to space constraints, these studies are summarized in Tables 1, 2, 3.

**TABLE 1:** BCI systems for consciousness and psychiatric disorders.

Year	Researcher	Disease	Patient/animal	Neurosensing mechanism	Stimulation modality	Decoding strategy	Effect	Ref
2024	Andrade et al.	Alzheimer's disease (AD)	Older adults with subjective memory impairment but without neurological or psychiatric disorders	Noninvasive EEG acquisition: 5 electrodes (Fz, Cz, Pz, C3, and C4) are used to record resting‐state electroencephalogram signals with a sampling rate of 500 Hz. The focus is on analyzing gamma band synchronization (35–45 Hz), PAF, and TBR. Preprocessing includes noise removal, band filtering (theta 4–8 Hz, beta 13–25 Hz), and epoch segmentation (2 s per segment).	Group A forms a closed loop through “EEG signal acquisition–biomarker decoding–visual feedback regulation,” while Group B's feedback is unrelated to neural signals and is an open‐loop intervention.	Real‐time calculation of PAF, gamma synchronization (phase synchronization analysis), and TBR is performed. Changes in biomarkers are evaluated using linear discriminant analysis (LDA) and support vector machine (SVM) classifiers.	Gamma‐band synchronization was significantly improved after EEG‐NF training.	[158]
2023	Sefati et al.	AD	Male rats	The photomultiplier tube was used to record the ultra‐weak photon emissions (UPE) from the rat hippocampus, with a wavelength range of 300–700 nm and a sampling time of 1 s, and the photon emission intensity was analyzed after dark noise correction. Simultaneously, the concentration of malondialdehyde (a lipid peroxidation marker) and the activity of acetylcholinesterase (AChE) were detected to evaluate the oxidative stress and the status of the cholinergic system.	Open loop: It only involves passive monitoring and drug intervention using the UPE signal, without forming a closed‐loop connection.	Through Pearson correlation analysis, a quantitative relationship was established between UPE and MDA (r = 0.8552) and AChE activity (r = 0.7793). 73% of the UPE variation could be explained by MDA, and 60% by AChE. Behavioral tests (Morris water maze, novel object recognition) verified the correlation between memory function and UPE.	A photonic chip‐based BCI is proposed for more precise monitoring and diagnosis of AD.	[159]
2018	Annen et al.	Disorders of consciousness (DOC), including unresponsive wakefulness syndrome (UWS) and minimally conscious state (MCS)	12 adult DOC patients, all unable to respond to commands at the bedside	8‐Channel EEG electrodes were used to record the P3 event‐related potential (ERP) evoked by tactile stimulation. Mechanical vibration stimulation (225 Hz, 30 ms) was applied to the left and right wrists and the right foot, with an interval of 270 ms between each stimulation, for a total of 480 stimulations.	Open loop: Visual feedback displays the BCI classification results, but the stimulation parameters are fixed, and there is no mechanism for adjusting the stimulation in real time based on the brain signals.	Using LDA to classify abnormal stimulation trials, extract ERP features from 100 to 600 ms, and conduct 10‐fold cross‐validation to calculate the BCI performance (with an accuracy rate >70% considered effective).	One MCS patient showed signs of “covert command following” in the VT3 paradigm.	[160]
2018	Spataro et al.	DOC (UWS)	Thirteen UWS patients and six healthy controls	Using 8‐channel EEG electrodes to record the P300 potential induced by vibrotactile stimulation, the stimulator was placed on the left and right wrists as well as the back. The frequency was 225 Hz, lasting for 30 ms, with a stimulation interval of 270 ms. Two paradigms were included: VT2 (two stimulation sources) and VT3 (three stimulation sources).	Open loop: The system displays the real‐time classification results of the brain waves, but the stimulation parameters remain constant and are not adjusted dynamically according to the brain signals.	Based on the LDA classification target and non‐target stimuli, ERP features within the range of −100–600 ms were extracted. The classification accuracy was calculated through 10‐fold cross‐validation, and the threshold was set at ≥50% as an effective response.	Four UWS patients showed clear EEG‐based measures of the task following one or both paradigms.	[161]
2024	Brewe et al.	Autism spectrum disorder (ASD)	Twenty‐seven male adolescents and adults with autism between the ages of 16 and 29 years	Using a 32‐channel wireless EEG cap to record the brain signals in the frontal‐parietal region (such as Fz, Cz, etc.) in real time, with a sampling rate of 256 Hz, to capture the neural activities during the facial emotion recognition (FER) process. Simultaneously, the MR head‐mounted device (Microsoft HoloLens) is used to present virtual avatars, demonstrating 6 basic emotions.	Closed loop: When the EEG features indicate that the emotion has not been successfully recognized, the system automatically increases the intensity of the virtual avatar's emotional expression (gradually increasing from 0% to 100%) until a successful brainwave signal is detected.	The EEG signals were classified in real time using the multivariate pattern analysis (MVPA) method. The brain electrical features (such as the signal patterns within 3000 ms) related to the correct FER were identified through the logistic regression model. After the model was trained to achieve a prediction accuracy of ≥85%, it was used for intervention.	No reliable improvement or decline for FER assistant participants	[162]
2019	Pereira et al.	ASD	5 ASD patients with fluent language and no intellectual disability	The bilateral fusiform face area (FFA) blood oxygenation level‐dependent (BOLD) signals were collected using a 1.5T magnetic resonance imaging (MRI). The hemodynamic changes were captured in real time through gradient echo planar imaging (EPI), with a spatial resolution of 3.2 × 3.3 × 4 mm^3^ and a temporal resolution of 1.5 s.	Closed loop: Through an MRI‐compatible visual feedback system (such as a thermometer bar graph), the activation level of the FFA is converted into a visual signal, guiding the participants to actively enhance FFA activity, forming a “signal acquisition–decoding–feedback” closed loop.	Based on the BOLD signal changes at the voxel level, a classifier was constructed using MVPA to distinguish FFA activation from the baseline state and to calculate the activation amplitude in real time.	Individuals with TD and ASD achieved FFA upregulation with contingent feedback.	[163]
2021	Eldeeb et al.	ASD	Twenty‐one individuals with autism	Using a 24‐channel EEG (following the international 10–20 system, with emphasis on collecting frontal lobe channels such as F3, Fz, and F4), with a sampling rate of 300 Hz and a band‐pass filter (0.1–40 Hz), the brain electrical signals during the presentation of visual feedback were recorded in real time.	Closed loop: A card game is designed based on the effective Posner task. The feedback is divided into WIN (correct, +10 points) and LOSE (wrong/too slow,−10 points). It is presented through a visual interface, with fixed stimulation parameters and no real‐time adjustments.	Extract time‐domain features (P300, feedback‐related negative wave (FRN), and late positive potential LPP) and frequency‐domain features (θ, *α*, *β* frequency band power, and total power of the frontal lobe), and select features through the weighted sequential forward selection (WSFS) algorithm. The SVM classifier is used to achieve sub‐trial classification.	The EEG‐based BCI system can effectively identify the differences in brain activity of ASD individuals under stress and non‐stress states and can be used as a tool for emotional regulation intervention to help ASD individuals learn and practice emotional regulation strategies.	[164]
2018	Fan et al.	ASD	Twenty autistic individuals aged 12 to 21 years	Using a 14‐channel wireless EEG device, electrode signals from the frontal lobe and temporal‐parietal regions, such as AF3, F7, and F3, were collected. The sampling rate was 128 Hz, with a band‐pass filter (0.2–45 Hz), and the virtual reality (VR) driving events and therapist ratings were synchronously recorded.	Open loop: In the VR driving environment, the difficulty of the task is adjusted through visual navigation, audio cues (such as “turn left”), and error feedback (such as “too fast speed”), but the stimulus parameters (such as vehicle density, and weather) are preset and not adjusted in real time according to the EEG.	Extract five types of features: statistical features, fractal dimension (FD), high‐order cross (HOC), band power (δ/θ/*α*/*β*/*γ*), and 2 Hz bin power; select features through weighted sequential forward selection (WSFS); and classify using the k‐nearest neighbor (kNN) algorithm.	EEG data can effectively identify the emotional state and cognitive load of autistic patients in driving tasks, especially the recognition accuracy of participation, pleasure, and defeat, which provides a basis for the development of EEG‐based passive BCI systems.	[165]
2019	Lim et al.	Attention‐deficit hyperactivity disorder (ADHD)	172 children between 6 and 12 years old were diagnosed with ADHD.	Using a dry electrode EEG headband (frontal Fp1/Fp2 and parietal Pz electrodes), 4–36 Hz frequency band brain signals (theta, alpha, beta1, and beta2 waves) were collected and then transmitted in real time to the computer via Bluetooth.	Closed loop: Visual feedback is achieved through the 3D game “Cogoland”: the higher the attention score, the faster the virtual character moves. Every two weeks, there is a 10‐minute academic task (English/mathematics questions) interspersed to enhance the application of real‐world scenarios.	By using machine learning models to analyze multi‐band EEG signals, a personalized “optimal attention” brainwave pattern is constructed. The attention/non‐attention states are classified in real time, and an attention score ranging from 0 to 100 is generated.	The BCI attention training program improved inattention symptoms in children with ADHD compared to untreated controls. Clinician‐rated ADHD‐RS inattention symptoms decreased by 3.5 points in the intervention group (SD 3.97) compared to 1.9 points in the wait‐list control group (SD 4.42) after 8 weeks.	[166]
2018	Qian et al.	ADHD	Sixty‐six boys with ADHD were randomly divided into an intervention group (44) and a control group (22)	Using a dry electrode EEG headband (frontal Fp1/Fp2 and parietal Pz electrodes), 4–36 Hz frequency band brain signals (theta, alpha, beta1, and beta2 waves) were collected and then transmitted in real time to the computer via Bluetooth. Combining EEG with RS‐fMRI, analyze functional connectivity (FC) and graph theory indicators (such as node centrality, and clustering coefficient).	Closed loop: Visual feedback is achieved through the 3D game “Cogoland”: the higher the attention score, the faster the virtual character moves. Every two weeks, there is a 10‐minute academic task (English / mathematics questions) interspersed to enhance the application of real‐world scenarios.	By using machine learning models to analyze multi‐band EEG signals, a personalized “optimal attention” brainwave pattern is constructed. The attention/non‐attention states are classified in real time, and an attention score ranging from 0 to 100 is generated.	Children in the intervention group showed significant improvement in inattention symptoms compared to the control group. The topological structure of the brain functional network was changed in the intervention group as follows. Ventral attention functional connections within the subventricular nucleus (SVN) and between the SVN and other networks were reduced. Local function processing was reduced in task‐active networks and the default mode network (DMN). Brain network topologies shifted from more regular to more random patterns.	[167]
2016	van der Kol et al.	Chronic post‐traumatic stress disorder (PTSD)	52 treatment‐refractory chronic PTSD patients (i.e., had received at least six months of trauma‐focused therapy without sustained clinical improvement)	Using the EEGer software and the Procomp2 amplifier, EEG signals from the T4 (active site), P4 (reference site), and the left ear (grounding point) were collected through dry electrodes. The power in the 2–6 Hz (slow wave), 22–36 Hz (fast wave), and 10–13 Hz (alpha wave) frequency bands was specifically analyzed.	Closed loop: Visual feedback is provided through computer games such as “Pac‐Man” and “Space Race.” When the target frequency band power reaches the standard, the game character's movement speed increases, accompanied by audio rewards. Patients need to actively maintain the target brainwave state to trigger the feedback. The threshold is adjusted every 3 min according to the patient's condition.	Real‐time suppression of power in the 2–6 and 22–36 Hz frequency bands, enhancement of power in the 10–13 Hz frequency band, generation of personalized thresholds through machine learning algorithms, and dynamic adjustment of feedback parameters.	After treatment, fewer PTSD patients in the NF training group (27.3%) met the diagnostic criteria for PTSD compared to the control group (68.2%). The NF group showed significantly better improvements in tension‐relieving activities, mood dysregulation, and mood instability than the control group. NF led to significant improvements in PTSD symptoms and emotional regulation among patients with chronic PTSD compared to controls. NF is expected to be an effective way to treat PTSD and improve emotional regulation.	[168]
2021	du Bois et al.	PTSD	29 PTSD patients in the Democratic Republic of the Congo (DRC), 9 in the control group, 10 receiving neurofeedback (NF) training, and 10 receiving motor imagery (MI) training	Neurofeedback (NF) group: Utilized an 8‐channel FlexEEG headband to collect EEG signals from electrodes such as Pz, with a focus on analyzing the power in the alpha frequency band (8–12 Hz). The reference electrode was placed on the right earlobe, and the ground electrode was located at AFz. MIgroup: Employed the FlexEEG headband to collect sensor motor rhythms (SMR, 8–13 Hz mu rhythm, and 13–30 Hz beta rhythm) from channels such as FC3‐CP3 and FCZ‐CPZ. Brain electrical patterns were analyzed through four standard frequency bands (alpha, low beta, high beta, and low gamma).	Closed loop: Both groups of studies achieved a closed‐loop BCI system through real‐time EEG signal processing and dynamic feedback regulation. The NF group focused on regulating alpha power. The MI group centered on decoding through motor imagery.	NF group: Real‐time calculation of alpha power of the Pz electrode, compared with the baseline, a threshold is set, and the adjustment effect is fed back through the game interface. A sliding window (500 ms) is used to dynamically update the alpha power threshold, adapting to individual differences. MI group: Uses the Filtered Common Spatial Pattern (FBCSP) combined with Mutual Information (MI) feature selection, classifies the left and right movement imagination patterns through LDA, optimizes the decoding accuracy based on six‐fold cross‐validation, and generates left and right movement instructions in real time.	Low‐cost, wearable neurotechnologies such as FlexEEG can effectively deliver NF training and reduce symptom severity in people with PTSD. NF training may improve PTSD symptoms through alpha‐wave rebound effects and improved brain network function. NF training could become a viable option for PTSD treatment in the DRC and other developing countries. The MI group showed no clinically relevant improvement in symptoms.	[169]
2021	Huang et al.	DOC	Seven patients with DOC, vegetative state (VS), or MCS	The 9‐channel EEG (CPz, P7, P3, Pz, P4, P8, O1, Oz, O2) was collected using the NuAmps equipment. The reference electrode was placed at the right mastoid, and the ground electrode was located at Fpz. The visual stimulus was two flashing “yes/no” squares on the left and right sides. The left square flashed at a frequency of 6.0 Hz and the right one at 7.5 Hz, inducing SSVEP; the square borders flashed randomly (200 ms appearance + 800 ms interval), inducing P300.	Closed loop: Based on the fusion result showing a “Yes/No” answer, a sliding window (6 s, 3 flashes) provides visual feedback to dynamically adjust the acquisition duration. Through Bayesian inference, dynamic intention determination is achieved for asynchronous control, eliminating the need for fixed intervals and reducing fatigue (with an average of 3.87 flashes per cycle, 21% fewer acquisition cycles compared to the synchronous system).	P300 Detection: Extract EEG signals within 0–800 ms after the square frame flashes, apply 0.1–20 Hz band‐pass filtering, downsample to 50 Hz, and use SVM to classify target/non‐target stimuli. SSVEP detection: use canonical correlation analysis (CCA) to calculate the correlation between EEG and reference signals (sine/cosine waveforms) and identify the flashing frequency (6.0 Hz or 7.5 Hz). Dynamic Fusion: Update the probabilities of P300 and SSVEP through a Bayesian framework, use linear SVM to combine the scores, and stop data collection when the posterior probability exceeds the threshold.	All healthy subjects achieved significant accuracy (72 –100 percent) using both the hybrid system and the single‐modal system. Hybrid asynchronous BCI systems are superior to systems based only on P300 or SSVEP. In addition, the study used an asynchronous method to dynamically collect EEG signals, which reduced the average number of rounds required by 21% and the online experiment time by 105 s compared to a synchronous system. The hybrid asynchronous BCI system may serve as a useful bedside aid for simple communication with DOC patients.	[170]
2025	Zhou et al.	Bipolar disorder (BD)	105 BD patients with self‐reported cognitive decline	Using 3.0T fMRI to collect brain structural and functional images, the functional connections between the V1 area and the dorsolateral prefrontal cortex (DLPFC) were located; the stimulation target was the V1 area (coordinates 6, −63, 15), based on the functional connection map of the DLPFC seed point of healthy individuals. Transcranial direct current stimulation (tDCS) used a current of 2 mA, with electrodes placed at PO3, FT7, and other sites, lasting for 20 min; repetitive transcranial magnetic stimulation (rTMS) used 10 Hz, 110% resting motor threshold, with 3000 pulses each time, located through the Black Dolphin navigation robot.	Simple stimulation: The stimulation parameters are preset (tDCS current, rTMS frequency/intensity are fixed), with no real‐time brain signal feedback regulation; the therapeutic effect is evaluated only through scales (THINC‐it, HDRS‐17), without closed‐loop interaction.	Offline analysis of fMRI data was conducted, where the centrality (DC) and clustering coefficient (NC) of brain network nodes were calculated using graph theory to evaluate the functional connectivity changes in the V1 area; real‐time brain electrocoding was not involved.	Patients who received both real rTMS and real tDCS showed significant improvements in symbol check items (time and accuracy) as well as symbol check accuracy. Patients who received real rTMS and real tDCS, as well as sham rTMS and real tDCS, demonstrated significant improvements in depressive symptoms. fMRI data showed increased cuneate gyrus activity in patients receiving both real rTMS and real tDCS. The combination of rTMS and tDCS targeting the V1 region may be a potential therapeutic strategy to improve cognitive impairment and depressive symptoms in patients with BD.	[171]
2021	Mishra et al.	ADHD	22 children with ADHD	EEG acquisition: Using a 64‐channel BioSemi system with a sampling rate of 1024 Hz, the signals were mapped to the prefrontal cortex (dorsal attention network) and the ventral visual cortex ROI through LORETA source localization technology. Target signal: The coherence of the α band (8–14 Hz) in the prefrontal cortex and the visual cortex during the 0–0.5 s cue period was calculated frequency‐specific synchronization (FSS). A decrease in FSS was positively correlated with the performance of the attention task.	Closed loop: Real‐time FSS decoding drives dynamic feedback. Users need to actively adjust their attention to change FSS, creating a two‐way interaction. Feedback parameters are adaptively adjusted according to the user's performance.	The FSS is calculated using the BCILAB tool, employing the multi‐taper coherence algorithm to map the FSS values onto a 0–100 point feedback scale (the lower the value, the stronger the desynchronization). The feedback threshold is dynamically adjusted based on the baseline FSS distribution. After each round of failure, the difficulty is reduced, and if there are consecutive successes, the challenge is increased.	Children with ADHD showed neuroplasticity in alpha‐wave FSS after cognitive neurofeedback (cNF) training and improved reaction time on sustained attention assessments. This study is the first to demonstrate the effectiveness of cNF for improving attention, providing new ideas for the application of cNF to treat diseases such as ADHD.	[172]
2022	Lin et al.	Schizophrenia	31 patients with schizophrenia or schizoaffective disorder	Using a 10‐channel wireless dry electrode EEG headband (Cognionics Quick‐10r), the coherence of the *γ* frequency band (30–50 Hz) at the F3 and F4 electrodes was collected, with a sampling rate of 500 Hz. The initial threshold was set based on the 20th percentile of the first minute of EEG data to ensure that the *γ* coherence exceeded the threshold for 80% of the time.	Closed loop: Play the video when the *γ* coherence exceeds the threshold; otherwise, pause it. The feedback signal is updated every second. Users need to actively regulate their neural activity to maintain the feedback.	Real‐time calculation of the *γ* coherence of F3–F4: Using the mscohere function in MATLAB, with a 3‐second sliding window and a 1‐second step size. Dynamic threshold update: Adjusted every 3 min based on the current coherence. If 80% of the time is above the threshold, the threshold is raised; otherwise, it is maintained or lowered.	EEG Neurofeedback (EEG‐NFB) effectively enhanced frontal gamma‐wave activity and improved the working memory performance of patients with schizophrenia. This study provides support for the application of EEG‐NFB technology to treat schizophrenia, and the developed BCI system provides a new tool for the implementation of gamma‐wave NFB training.	[173]

**TABLE 2:** BCI systems for partial movement disorders.

Year	Researcher	Disease	Patient/animal	Neurosensing mechanism	Stimulation modality	Decoding strategy	Effect	Ref
2020	Ganzer et al.	Spinal cord injury (SCI)	A 27‐year‐old male with stable non‐spastic C5 paraplegia	A 96‐channel Utah microelectrode array (MEA) was implanted into the left primary motor cortex (M1) to collect multi‐unit neural activities ranging from 70 to 3000 Hz.	Closed‐loop system: The right biceps brachii is equipped with a vibration tactile array, which provides real‐time feedback of the decoded tactile information (the vibration intensity is dynamically adjusted according to the grip force).	Use the SVM)classifier.	Multimodal BCI significantly improved motor control. With the vibrotactile feedback system, patients were able to perceive the tactile sensation of objects more accurately, and the tactile recognition rate increased from the random level to more than 90%.	[174]
2023	Luo et al.	Amyotrophic lateral sclerosis (ALS)	A 61‐year‐old male with severe motor neuron disease (ALS), severe motor aphasia, and dysphagia but still able to speak and enunciate	Two 64‐channel electrocorticography (ECoG) arrays were implanted in the ventral sensorimotor cortex to collect high gamma energy (HGE) signals ranging from 70 to 170 Hz.	Closed loop: Visual feedback (color changes on the communication board) and functional control (operation of devices such as lights and televisions).	A convolutional neural network based on the InceptionTime architecture classifies six types of voice commands using HGE features (a window of the first 2 s of peaks + the last 0.5 s).	Users were free to issue voice commands at their own pace. The system maintained high decoding accuracy (median accuracy: 90.59%) and fast response for up to 3 months.	[175]
2024	Séguin et al.	Locked‐in syndrome (LIS) and complete LIS (CLIS)	Three patients with LIS and four patients with ALS	A 32‐channel EEG (ActiCap system) was used to record the scalp electrical activity, with a particular focus on collecting ERPs such as P300 and N200 in the auditory‐related brain regions.	Closed loop: Visual feedback (such as “Select answer as ‘yes.”) is presented in real time. The subject can adjust their attention based on the feedback.	The xDAWN algorithm is used for spatial filtering, combined with a supervised probabilistic classifier, to classify “yes/no” responses based on ERP features (such as P300) of standard sounds and deviant sounds.	Online BCI accuracy in healthy subjects averaged 86%, with 10 achieving accuracy above 90%. Most patients were unable to achieve online performance above random levels, and two ALS patients achieved 100% accuracy.	[176]
2024	Zou et al.	Epilepsy	Male Sprague‐Dawley rats (200–220 g)	Wireless EEG records the brain's electrical signals of the hippocampus and extracts features such as coastline (CL) and standard deviation (STD).	Closed loop: 250 kHz ultrasound, optimized to a pulse frequency of 20 Hz, an acoustic pressure of 0.9 MPa, and percutaneous stimulation of the vagus nerve. Adjust dynamically until seizure suppression is achieved.	The multi‐level threshold model determines an episode when the EEG features exceed the threshold and triggers the ultrasound.	The acoustic brain–computer interface (aBCI) system decodes seizures in real time and controls seizures by ultrasound stimulation of the vagus nerve. aBCI had a stronger antiepileptic effect than conventional electrical vagus nerve stimulation in acute experiments.	[177]
2022	Liu et al.	Epilepsy	Five patients with intractable epilepsy	Eight to twelve contact SEEG electrodes were implanted in the MT area. Using Laplace/bipolar differential reference technology, ERPs ranging from 1 to 20 Hz and high gamma signals ranging from 60 to 140 Hz were collected.	Closed loop: Visual feedback displays the spelling characters in real time. When the user focuses on a specific row/column, it triggers a visual motion stimulus, forming a “attention–neural signal–decoding–feedback” closed‐loop regulation.	The logistic regression classifier combines ERP and high‐gamma features to distinguish between target/non‐target stimuli, and the dynamic stopping algorithm optimizes the decision‐making time based on the classification confidence.	Both offline and online tests yielded satisfactory results, with a maximum spelling speed of 62 bits/min (12 characters/min).	[178]
2022	Chen et al.	Parkinson's disease (PD)	One patient with PD	DBS electrodes are implanted in the bilateral subthalamic nuclei (STN), and 8‐channel local field potentials (LFP) are simultaneously collected. Surface electromyography (EMG) and accelerometers are used to synchronously record motor signals.	Closed loop: Visual feedback drives the control of the virtual wheelchair: upper limb movements trigger the selection of turns, lower limb movements control the forward movement, and the rest state stops. Patients adjust their movements through the screen feedback.	Extract the *α* (10–13 Hz), *β* (13–35 Hz), and *γ* (35–50 Hz) frequency domain features of LFP and use wavelet transform and a single‐hidden‐layer recurrent neural network to classify the rest / upper limb / lower limb motion states.	Postoperative symptoms were significantly improved, and the machine learning classifier effectively decoded movement status. The patient successfully completed the two‐dimensional task by controlling the virtual wheelchair with limb movement.	[179]
2022	Kramer et al.	Epilepsy	Thirteen patients with intractable epilepsy	High‐density mECoG grids were implanted in the S1 hand area, and somatosensory perception was triggered through bipolar stimulation.	Open loop: The stimulation parameters are adjusted by the epileptologist based on clinical safety, without a real‐time closed‐loop feedback mechanism.	Based on the Penfield somatosensory mapping, the mapping relationship between the stimulating electrodes and the anatomical regions of the hand was analyzed. The coverage area, redundancy (the number of electrode pairs per region), and resolution (the number of covered regions per electrode pair) were calculated.	The mini ECoG grid can effectively stimulate the somatosensory cortex and produce a perceptible somatosensory experience. The mini ECoG mesh has low redundancy and high resolution, making it an ideal choice for somatosensory BCI systems.	[180]
2021	Patel et al.	Epilepsy	Eleven epileptic patients receiving diagnostic depth electrode implantation	Deep electrodes were implanted into the limbic system (such as the hippocampus and amygdala) and memory‐related brain regions of epilepsy patients. The activities of individual neurons were recorded, and the single neuron discharge sequences were extracted using the KlustaKwik toolkit.	Closed‐loop visual feedback: The position of the red square changes in real time according to the discharge rate of the target neuron. When it exceeds the threshold (the white line), it changes color to purple, indicating success. The difficulty of the task is dynamically adjusted based on the performance.	Apply a 200 ms Gaussian smoothing to the firing rate of a single neuron to generate a control signal, which is mapped to the vertical movement of the red square on the screen. Define “learner” as the participant whose single neuron firing rate significantly increases with the number of training sessions (with a positive slope in linear regression), and analyze the changes in the specificity and synchrony of their neural activities.	Learners were able to modulate the activity of target neurons with high specificity, and there were significant increases in firing rate and burst frequency.	[181]
2019	Kokkinos et al.	Focal epilepsy	Eleven patients with focal epilepsy treated with responsive neurostimulation (RNS)	Implant the RNS system, which uses 4‐channel bipolar ECoG electrodes to continuously record the cortical electrical activity in real time. Capture the seizure patterns (ESPs) and identify the onset point of the seizure for spectral analysis.	Closed loop: Real‐time detection of electrical stimulation triggered after an epileptic seizure, with parameters adjusted according to clinical needs. Feedback is recorded through ECOG after the stimulation, forming a “detection–stimulation–feedback” closed loop.	Define the direct effect (the immediate change within 5 s after the stimulus) and the indirect effect (the change that occurs after 10 s or more or is not time‐limited). Analyze the correlation between the electrophysiological effect and the clinical outcome through Fisher's exact test.	Two main modes of electrophysiological regulation were found: direct and indirect. Direct effects included seizure suppression and early frequency modulation, but clinical outcome improvement was not associated with this mode. Indirect effects occurred outside the triggering stimulus event and included spontaneous seizure suppression, modulation of frequency, fragmentation, and modulation of seizure duration. These effects were associated with improved clinical outcomes.	[182]
2024	Köhler et al.	PD	25 PD patients (12 females)	Implant bilateral STN‐DBS electrodes and unilateral subdural ECoG electrodes to record cortical electroencephalogram (ECoG) and LFPs of the subthalamic nucleus (STN‐LFP). Simultaneously record electromyogram (EMG), accelerometer, and action trigger signals (button press/wrist rotation).	Closed loop: Adjust the STN‐DBS contacts based on clinical outcomes. Open loop: Take levodopa orally, and systematically regulate neurotransmitters.	Based on LDA, a classifier was trained to decode motor intentions through the seven frequency band features (*θ*, *α*, *β*, *γ*, etc.) of ECoG/STN‐LFP, and the latency from intention to execution was calculated. The time‐reversed Granger causality analysis (TRGC) was used to quantify the coupling direction and frequency characteristics of the cortical–subthalamic nucleus oscillations.	In the low dopaminergic state, the latency between motor intention and motor execution was longer. Both dopamine drugs and DBS significantly shortened these latencies. Both dopamine drugs and DBS shifted the cortico‐subthalamic nucleus oscillatory information flow from the antimotor beta frequency to the promotor theta frequency.	[183]
2021	Arlotti et al.	PD	Three patients with PD, all previously treated with DBS	The implanted pulse generator (IPG) records the LFPs of the STN through symmetrical/asymmetrical electrode configurations and simultaneously suppresses the stimulation artifacts in real time.	Closed loop: In the adaptive mode, the stimulation amplitude is linearly adjusted according to the beta power, forming a “neural signal–decoding–stimulation” closed loop.	Based on the beta frequency band (10–35 Hz) power of LFP as a biomarker, the motion intention and pathological state are decoded in real time through the LDA classifier. The closed‐loop algorithm is embedded in the IPG firmware, and the stimulation amplitude is dynamically adjusted according to the beta power.	The AlphaDBS System provided reliable and artifact‐free LFP recordings. The AlphaDBS System successfully achieved closed‐loop control and dynamically adjusted the stimulation parameters according to the *β*‐wave power. The AlphaDBS system can be used for real‐time monitoring and research analysis.	[62]
2022	Lazarou et al.	Neuromuscular disease (NMD) SCI PD	Thirty participants, 19 with NMD, 10 with SCI, and 10 with PD.	The SMI eye‐tracking system is used to capture the movement trajectories of the eyes, which are then converted into interface control signals in real time; EEG equipment records brain electrical activities to assist in identifying the user's intentions.	Closed loop: The system decodes the user's intentions in real time through eye movement/brainwave signals, triggers visual/auditory feedback, and the user adjusts the interaction strategy based on the feedback, forming a two‐way regulatory link.	The eye movement signals are converted into operation instructions, such as clicks and scrolls, through threshold detection and pattern recognition; the electroencephalogram signals are classified by the LDA classifier to distinguish between the focused state and the fatigued state, optimizing the interface response; an internal dynamic adjustment algorithm is built in, automatically optimizing the eye movement sensitivity according to the user's operation efficiency.	The platform appeared to have a greater positive effect on social integration measures of NMD participants. Some SCI participants improved their social skills but, at the same time, reported fatigue during platform use. Some PD patients were excited about the platform and saw it as having a positive effect on their interests, but others were anxious and resistant.	[184]
2024	Caffi et al.	PD	An adult male with PD developed resting tremor and bradykinesia of the right hand in his 40s.	The AlphaDBS device records LFP through bilateral STN electrodes.	Adaptive deep brain stimulation (aDBS) mode: When the *β* amplitude exceeds the threshold, the stimulation current is reduced; when it is below the threshold, the current is increased, forming a closed loop. Conventional deep brain stimulation (cDBS) mode: Fixed current, without real‐time feedback.	Using a linear algorithm, the stimulation current is dynamically adjusted based on the exponential moving average of the *β*‐band LFP amplitude, with the adjustment range limited within the clinically effective range. The LFP spectrum is decomposed using a Gaussian function to separate the periodic oscillations from the non‐periodic components, and the peak frequency and amplitude of the *β*‐band are extracted.	aDBS was more effective than traditional cDBS for controlling PD motor symptoms. Patients with aDBS had higher *β*‐wave amplitudes and greater fluctuation in the basal ganglia. Under aDBS, the *β*‐wave amplitude was lower during sleep than during wakefulness.	[63]
2022	Merk et al.	PD	Eleven PD patients receiving DBS surgery	During the operation, simultaneous recordings were made of the sensorimotor ECoG (cortical hand area) and STN‐LFP from 11 patients with PD, with a sampling rate of 1000 Hz, and 8 frequency band features (*θ*, *α*, *β*, etc.) were extracted.	Open loop: There is no real‐time stimulation feedback. Only offline analysis is conducted to determine the correlation between the decoding results and clinical indicators (UPDRS‐III).	Using models such as gradient boosting decision tree (XGboost), elastic net, and neural network, we integrate multi‐timepoint features within 500 ms (a 40‐dimensional feature vector). Bayesian optimization is employed to optimize the hyperparameters, and the model performance (*R* ^2^ coefficient) is evaluated through nested cross‐validation.	The ECoG signal was more accurate than the STN‐LFP in decoding grip force. The XGBoost model performed best in decoding grip strength. Decoding performance was negatively correlated with the degree of motor impairment. Low‐frequency *β*‐wave bursts in the subthalamic nucleus were correlated with the degree of dyskinesia and negatively with the performance of ECoG based on grip strength. Whole‐brain connectomics can predict the decoding performance of ECoG signals.	[185]
2024	Jochumsen et al.	PD	Nine male patients with PD	Recorded 64‐channel EEG (including sensors such as F3, Fz, and Cz in the motor area) and right forearm/anterior tibial muscle EMG from nine Parkinson's patients. The sampling rate was 512 Hz. Signals in the frequency range of 0.1–30 Hz were extracted. The onset of movement was determined by EMG, and the EEG was segmented into epochs from 2 s before the movement to 1 s after the movement. At the same time, resting‐state EEG was collected as a control.	Open loop: Only offline analysis of the correlation between EEG and motor intention was conducted, without involving real‐time stimulation regulation or closed‐loop control. The stimulation was merely a visual cue in the experimental design, without neuro‐signal‐driven stimulation feedback.	Feature extraction: Time domain (mean values of different time periods before movement), template matching (cross‐correlation with the average waveform of the training set), and spectrum (power spectral density of 8–30 Hz). Classifier: Random Forest (RF), LDA, kNN	BCI based on MRCP effectively detected the motor intention of patients, providing a new idea for the development of PD‐assistive technologies.	[186]
2023	Sindhu et al.	Intractable epilepsy	Three patients (1 female and 2 males, aged 15.4, 11.9, and 19 years, respectively) with intractable epilepsy.	Implant 8 × 8 high‐density subdural grid electrodes, simulating a larger surface area by short‐circuiting adjacent electrodes through electricity. Record intracranial electroencepholography (iEEG) from 3 patients with refractory epilepsy, focusing on the epileptic discharges during interictal periods and analyzing the signal characteristics after filtering.	Open loop: There is no feedback loop for stimulation. It only records natural brain electrical activity and does not involve the adjustment of stimulation parameters or closed‐loop control.	Feature analysis: Calculate RMS amplitude, power spectral density (1–100 Hz), inter‐channel correlation (low frequency, Gamma band), and SNR. Statistical methods: Wilcoxon rank sum test and depth permutation test to compare the differences in signals from different electrode sizes.	With increasing electrode size, iEEG amplitude and power decreased, while the interchannel correlation did not change significantly. The SNR of epileptic spikes was generally highest in the smallest electrodes, but 39% of spikes had the highest SNRs in the larger electrodes.	[187]
2022	McNamara et al.	PD	Lister hooded rat model of PD (by 6‐hydroxydopamine [6‐OHDA] lesion)	Record the ECoG of the motor cortex of Parkinsonian rats; track the phase of the *β* waves (35–40 Hz), in real time; and synchronously apply electrical stimulation to the globus pallidus or subthalamic nucleus. Stimulation parameters: Bipolar charge‐balanced pulses (50–70 µA, 95 µs pulse width), triggered based on real‐time phase estimation.	Closed loop: The system triggers stimulation through real‐time phase tracking, and changes in brain activity, and then feed back to regulate the stimulation mode, forming a stable interactive balance.	Using the OscillTrack algorithm, it directly processes broadband signals. Through iterative matching of sine/cosine reference waves, zero‐delay phase estimation is achieved, with a resolution of a single oscillation cycle. Under closed‐loop conditions, the stimulation phase is locked to a specific phase of the *β* wave (such as the ascending phase or the descending phase), and the closed‐loop stimulation pattern is replayed when in open‐loop mode.	Stable amplification or suppression of cortical beta oscillations was achieved by phase‐locked stimulation. The degree of amplification or inhibition depended on the target phase of the stimulus, and the stimulus pattern adjusted adaptively as the oscillatory activity changed. Amplification of *β* oscillations resulted in slower movement in rats, suggesting a functional effect of *β* oscillation modulation on behavior.	[188]
2024	Fang et al.	PD	/	Using the *β* frequency band (13–30 Hz) power of the medial pallidum or the subthalamic nucleus as the feedback signal, the real‐time power value is calculated through multi‐cue spectral analysis. Simulating non‐stationary neural dynamics: By dynamically adjusting the neural coupling strength parameter (pd), scenarios such as symptom relief, circadian rhythm changes, and stimulus‐induced plasticity can be simulated.	Closed loop: The system continuously tracks the phase and power of the *β* oscillation and dynamically adjusts the DBS frequency, thereby achieving a two‐way feedback regulation.	Construct an adaptive state space model, incorporating nonlinear terms and uncertain items to represent the dynamic characteristics of the neural system. Use the recursive least squares method to estimate the model parameters in real time, approximate the nonlinear functions through Taylor expansion, and track the non‐stationary changes.	Under different nonlinear and nonstationary neurodynamics, the robust adaptive DBS method was able to accurately regulate the basal ganglia Parkinsonian beta‐band oscillatory power with little control error, bias, or deviation. The proposed method demonstrated high accuracy across treatment targets and consistently outperformed conventional on/off and linear DBS methods.	[189]
2024	Fauser et al.	PD	Semi‐Parkinsonian rat model (6‐OHDA injury)	Implant electrodes in the bilateral subthalamic nucleus or subthalamic nucleus of the foot with continuous high‐frequency stimulation (for 5 weeks), without real‐time neural signal feedback monitoring. Mark the newly generated neurons by immunohistochemistry and quantify neurogenesis in the subventricular zone—olfactory bulb (OB) and dentate gyrus.	Open loop: Fixed parameter stimulation (frequency, pulse width, etc.), without dynamic adjustment, belongs to the open‐loop stimulation mode.	The process of decoding without neural signals involves only the analysis of neural changes through histological methods.	STN‐DBS consistently increased the number of newborn dopaminergic and gamma‐aminobutyric acid (GABA)ergic neurons in the olfactory bulb. This may be the mechanism by which DBS affects some of the non‐motor symptoms of PD (memory loss and olfactory dysfunction). Entopeduncular nucleus‐deep brain stimulation (EPN‐DBS) did not affect neurogenesis. STN‐DBS and EPN‐DBS may influence neurogenesis through distinct mechanisms.	[190]
2023	Saal et al.	Drug‐refractory epilepsy	Three drug‐resistant epilepsy patients with deep electrodes implanted for sEEG	Implantable stereoelectroencephalography (sEEG) electrodes record LFPs from the hippocampus, capturing power in the theta (4–8 Hz) and gamma (52–99 Hz) frequency bands, with a sampling rate of 1024 Hz. DIXI MicroDeep electrodes are implanted bilaterally in the hippocampus, with contact points ranging from 5 to 11.	Open loop: The output is the classification result of the vehicle's movement speed in the virtual environment, without real‐time neural stimulation regulation. The patient controls the virtual navigation task through the keyboard. The BCI is only used to decode the speed, and no closed loop has been formed.	Based on the LDA classifier, combined with theta and gamma power features, the virtual movement speed is decoded (to distinguish the fastest/slowest 10% speed intervals). The classification accuracy is evaluated using ten‐fold cross‐validation and the bootstrap resampling method. The AUC value ranges from 0.62 to 0.72.	The study is the first to demonstrate that motion speed can be decoded from human hippocampal signals, laying the foundation for the development of BCI technology based on hippocampal signaling. This technology can help paralyzed patients regain the ability to move independently, improving their quality of life.	[191]
2023	Levett et al.	SCI	21 patients with cervical SCI with injury levels including C4, C5, C4/C5, and C5/C6.	Implanted cortical microelectrode arrays(ICMA, such as the Utah array with 96 channels as the main type) or cortical electroencephalogram (ECoG) electrodes directly record the neural activities in the motor cortex, with sampling rates covering the high‐frequency characteristics of neural signals.	Closed loop: The outputs include non‐invasive functional electrical stimulation (FES, 47.6%, such as cuff electrodes), invasive FES (4.8%), or external devices (47.6%, such as mechanical arms). All studies achieved motor function recovery through the “neural signal recording–decoding–functional output–feedback” closed‐loop system.	Machine learning and statistical models perform real‐time decoding of neural signals: Support vector machines (SVM, 38.1%), linear estimators (33.3%), Hidden Markov models (14.3%), etc., map neural activity to motor intentions. The decoding parameters are adjusted based on real‐time neural signals to adapt to individual differences.	All patients regained motor function on assigned tasks, but there was heterogeneity in the findings and a lack of uniform clinical evaluation criteria. Invasive BCI techniques show potential in restoring motor function, but there is currently no technology that can fully restore autonomous function in patients.	[192]

**TABLE 3:** BCI systems for stroke and speech, hearing, and visual disorders.

Year	Researcher	Disease	Patient/animal	Neurosensing mechanism	Stimulation modality	Decoding strategy	Effect	Ref
2024	Wang et al.	Stroke	296 patients with ischemic stroke	Using an 8‐channel semi‐dry/semi‐wet electrode EEG cap (with positions such as FC5, Fz, C3, etc.), brain electrical signals during motor imagination were collected, with the impedance maintained at 60–100 kΩ. The FES stimulation signals and VR interaction data were simultaneously recorded to ensure real‐time correspondence between the feedback and the brain electrical signals.	Closed loop: Through real‐time brain electrical codes to drive FES and VR feedback, patients need to actively adjust their motor imagery to maintain the feedback.	Offline stage: Use the Public Space Pattern (CSP) and SVM to train the classification model, identifying the brainwave patterns corresponding to motor imagery (such as shoulder abduction, clenching the fist, etc.). Online stage: Use a sliding time window (2 s) to extract features in real time and classify them quickly through SVM, with decoding delay <0.1 s.	The BCI group achieved greater improvements in Fugl–Meyer assessment for upper extremity (FMA‐UE), action research arm test (ARAT), and Wolf Motor Function Test (WMFT) scores than controls. The incidence of adverse events was similar in the two groups.	[193]
2024	Ma et al.	Stroke	Forty‐six eligible stroke patients, 40 of whom completed the study	Using an 8‐channel semi‐dry/semi‐wet electrode EEG cap (with sites such as FC5, Fz, C3, etc.), the brain electrical signals of motor imagination were collected. Simultaneously, the interactive data of FES and VR were recorded to ensure that the feedback corresponded to the brain's electrical signals in real time.	Closed loop: The VR scene (such as “catching a butterfly”) is dynamically updated based on the decoding results. If the imagination is successful, a smiling face is displayed; if not, a crying face is shown. FES stimulates the corresponding muscle groups based on the decoding results (current ranging from 8 to 50 mA), and the stimulation duration matches the movement imagination cycle.	Offline stage: Using the Public Space Pattern (CSP) and SVM to train the classification model to identify movement imagination patterns such as shoulder abduction and fist clenching. Online stage: Real‐time extraction of features with a 2‐second sliding time window, SVM classification decoding delay < 0.1 s.	The upper limb motor function FMA‐UE score was significantly improved in the BCI group compared to the control group. The ipsilateral cingulate gyrus, precuneus, inferior parietal lobule, postcentral gyrus, middle frontal lobe, superior temporal gyrus, and bilateral cingulate gyrus showed significant activation when the BCI group performed the motor grasp task on the affected side. Activation levels of the ipsilateral superior frontal gyrus (medial) and middle frontal lobe while imagining the affected side grasping were higher in the BCI group after treatment. In the BCI group, zReHo values increased in the ipsilateral precuneus and contralateral cuneus but decreased in the contralateral middle temporal gyrus, temporal pole, and superior temporal gyrus. Increased FMA‐UE scores were positively correlated with mean zALFF in the contralateral precentral gyrus and mean zReHo values in the right cuneus.	[194]
2024	Brunner et al.	Stroke	Forty patients with severe upper limb paralysis	The 16‐channel EEG cap (International 10/20 system, with electrodes such as FC5, C3, etc.) was used to collect the brain electrical signals. The reference electrode was placed on the right earlobe, and the ground electrode was located at FPz. The impedance was maintained within a reasonable range.	Closed‐loop system: Brain electrical signals → Real‐time decoding → Trigger FES/visual feedback → Patient adjusts imagination → Signal re‐decoding, forming a two‐way interaction.	Offline stage: Train the classification model using CSP and SVM, and identify the movement imagination patterns of the left and right hands. Online stage: Real‐time calculation of the event‐related desynchronization (ERD) in the 8–30 Hz frequency band. Trigger feedback when the classification confidence is greater than 50%.	There was no significant difference in ARAT score improvement between the BCI training group and the control group. Cortical‐spinal tract (CST) integrity was a significant predictor of improved upper limb motor function, and CST integrity was more important for the improvement of upper limb function than the intervention received. During BCI training, there were significant changes in ERD and its lateralization in the affected hemisphere.	[195]
2023	Rocca et al.	Blindness	Three blind volunteers	Using a 100‐channel UEA (with a 4 mm × 4 mm substrate and 1.5 mm long needles), it was implanted in the V1/V2 area of the occipital lobe. It was connected to the skull base via gold wires, and the electrical activities of cortical neurons were recorded. Before the operation, the optimal implantation site was determined through transcranial magnetic stimulation (TMS), ensuring that the stimulation could induce reliable visual hallucinations (phosphenes).	Direct neural stimulation: Electrical pulses are sent to the microelectrodes through an external device to stimulate the occipital cortex and induce visual illusions. There is no closed‐loop feedback regulation. During the experimental stage, the stimulation parameters are adjusted based on the patient's subjective reports (such as the shape and color of the visual illusions).	There is no real‐time decoding process. During the experimental stage, the stimulation parameters (amplitude, frequency, pulse width) were optimized using an external device to induce stable optical hallucinations with the minimum current. After the surgery, the electrode positions were verified using CT and MRI to ensure the implantation accuracy (error < 1 mm).	All three volunteers successfully completed the surgical and experimental phases. Through training, the patient was gradually able to recognize visual information such as color, shape, and motion.	[196]
2025	Tankus et al.	Voice disorders	A 37‐year‐old male with drug‐refractory epilepsy was implanted with deep intracranial electrodes to identify epileptic foci.	Six deep electrodes (each containing nine micro‐wires) were implanted, targeting the left/right orbitofrontal cortex, left anterior cingulate cortex (LrAC/LdAC), and left/right hippocampus. High‐frequency neural activities (>250 Hz, including single neuron discharges) were collected. The electrode positions were determined through clinical assessment before the surgery to ensure no interference from epileptic foci.	Closed loop: Based on the decoding results, pre‐recorded vowel sounds are played. The patient optimizes the neural signals by adjusting the imagination strategy, and the system updates the prediction in real time, forming a two‐way interaction.	The high‐frequency activity rate within a 100 ms time window was extracted online, and a linear SVM) classifier was used. Based on 48‐channel features (baseline activity rate + peak activity rate), the vowels (/a/ and /e/) were predicted in real time. The offline training used overt speech data, and the test stage was applied to silent imagined speech. The decoding delay was 0.03 s.	The speech neuroprosthesis system based on rAC/MOF and hippocampal high‐frequency electrical activity decoding achieved high decoding accuracy and possibly induced neuroplasticity, suggesting a novel strategy for the development of speech‐restoring neuroprostheses.	[197]
2020	Lee et al.	Stroke	Twenty‐six patients who developed upper limb paralysis after stroke	The scalp electrical signals were collected using EEG, with electrodes placed at Fp1, Fp2, F3, F4, C3, C4, and other positions (in the International 10–20 system), and the reference electrode was placed at the left or right mastoid. The sampling frequency was 0.1–50 Hz. The signals were processed through band‐pass filtering and Laplace transformation. The ratios of sensory‐motor rhythm (SMR, 12–15 Hz), medium *β* waves (15–18 Hz), and *θ* waves (4–8 Hz) were calculated in real time as indicators of attention.	Closed loop: FES stimulates the extensor muscles of the wrist. The parameters are a frequency of 60 Hz, an intensity of 20–27 mA, and a pulse width of 150 µs. The trigger condition is when the brainwave signal meets the standard. Visual (screen prompts) and auditory (sound feedback) simultaneously inform the patient of the feedback result, forming a closed loop.	Real‐time calculation of the frequency spectrum of EEG signals. When the attention threshold exceeds the preset value (such as increased SMR and mid‐*β* waves), trigger FES. Features are extracted using the Fast Fourier Transform (FFT), and the SVM classifier is used to identify the movement intention.	The AOT + BCI‐FES group scored significantly better than the control group on the FMA‐UE, WMFT, motor activity log (MAL), and modified Barthel index (MBI). EEG parameters (alpha wave, beta wave, concentration, and activation) were also improved.	[198]
2022	Guo et al.	Stroke	Thirty patients with post‐stroke hand dysfunction	The EEG signals were collected using the Emotiv EPOC. The electrodes were placed at the P7, P8, O1, and O2 positions, with a sampling rate of 128 Hz. The visual stimuli consisted of flashing squares on the left and right sides (with frequencies of 12 and 15 Hz), which induced the SSVEP signals.	Closed loop: The screen displays real‐time animations of the robot glove's movements, corresponding to the flashing square that the user is looking at. The soft robot glove performs finger flexion and extension movements through pneumatic actuators, stimulating muscle groups such as the wrist extensor muscles.	The CCA algorithm is used for real‐time classification of EEG signals. The window length is 3 s, and a control command is triggered when the same frequency is detected in 4 consecutive windows. Only when the CCA correlation exceeds the preset value will an effective instruction be output.	SSVEP‐BCI‐controlled soft robot glove rehabilitation resulted in better hand function recovery than simple robot glove rehabilitation, and its efficacy was comparable to that of previously reported motor imagery brain–computer interface (MI‐BCI)‐based robotic hand rehabilitation.	[199]
2023	Lima et al.	Stroke	A 73‐year‐old patient with right hemiplegia after a stroke (subacute phase)	The brain electrical signals were collected using an 8‐channel EEG cap (with electrodes at FC1, FC2, C3, C4, etc., following the international 10–20 system), with the reference electrode placed on the left and right ears. The sampling rate was not specified. The tDCS used a current of 2 mA, with the anode placed on the primary motor cortex (M1) on the affected side and the cathode placed on the cerebellum on the opposite side. The stimulation lasted for 20 min.	Closed loop: In the VR scene, a first‐person avatar rides a tricycle, synchronizing with the electric pedal movement. The BCI decoding results drive the electric pedal to perform flexion and extension actions, stimulating the lower limb muscles. The patient regulates the brain signal through MI → the BCI decoding triggers the pedal movement → the VR visual feedback reinforces MI, forming a “brainwave–action–feedback” loop.	Real‐time processing of EEG signals is carried out based on the OpenViBE platform. Through band‐pass filtering (0.1–40 Hz) and CCA, μ and *β* rhythm changes related to MI are identified. During the calibration stage, the classification model is trained using resting state, MI, and passive pedal movement data. In the online stage, the pedal movement intention is decoded in real time.	DCS and MI‐BCI VR training improved lower limb functional recovery after stroke. tDCS enhanced cortical excitability and promoted neural plasticity. MI‐BCI and VR can provide an immersive training environment to improve patient engagement and training effects.	[200]
2025	Svejgaard et al.	Stroke	Twenty‐four patients with subacute stroke	Using a 10‐channel EEG cap (with electrodes at Fp1, Fz, C3, etc., following the international 10–20 system), movement ‐related cortical potentials (MRCP) were collected. The peak negative potential (PN) was specifically extracted as the marker for motor cortex activation. Simultaneously, the electromyographic (EMG) signals of the extensor carpi radialis longus (ECRL) muscle were recorded to assess the excitability of the corticospinal tract.	The system dynamically adjusts the timing of stimulation through real‐time EEG decoding. The patient's motor intention (MRCP) is strictly coupled with the peripheral feedback, forming a complete closed loop. The control group received open‐loop stimulation (with fixed stimulation intensity and no neural signal feedback).	Offline analysis of the PN latency of MRCP, calculation of the optimal triggering time for peripheral stimulation (PN time = 25 ms) to ensure that the incoming volley is synchronized with cortical activation. Band‐pass filtering (0.05–3 Hz) and a peak detection algorithm are used to identify PN.	After associative BCI training, patients in the intervention group showed a significant increase in motor‐evoked potential (MEP) amplitude that lasted for at least 30 min. There was no significant change in MEP amplitude among patients in the sham control group. Correlational BCI training enhanced corticospinal excitability in a limb. The results of this study offer the possibility of enhancing the effects of existing upper limb rehabilitation programs.	[201]
2023	Fateeva et al.	Poststroke cognitive impairment (PICK)	Thirty patients aged 22 to 82 years with ischemic stroke (duration no longer than 3 months) and moderate cognitive impairment (Montreal Cognitive Assessment [MoCA] score < 26).	Using an 8‐channel EEG head‐worn device, electrodes were placed at the Fp1, Fz, and other positions (in the International 10–20 system), with the reference electrode located at the right earlobe. The P300 ERP was recorded in real time. The screen displayed Russian letters, and the highlighting of the target letter triggered the P300 wave. The sampling rate was 1200 Hz.	Closed loop: When the user gazes at the highlighted letter, the system decodes it and displays the letter in the text box. The system processes the EEG signals in real time and dynamically adjusts the feedback. The patient controls the letter input through attention, forming a two‐way interaction.	Offline calibration stage: Using machine learning to identify individual P300 waveform characteristics and determine the peak latency and amplitude thresholds. Online stage: Real‐time analysis of EEG signals. When the P300 wave (positive potential with a 300 ms latency) is detected, trigger the target letter input feedback.	Patients in the experimental group exhibited a significant improvement in MoCA “attention” domain scores. Patients in the experimental group achieved a significant increase in the number of letters entered during daily tasks using the BCI over 10 days. Scores on the MoCA “attention” domain were positively correlated with performance in the experimental BCI group.	[202]
2024	Kim et al.	Stroke	27 chronic stroke patients with weak wrist extensor strength (Medical Research Council wrist extensor [MRC‐WE] ≤ 2)	A 16‐channel EEG (International 10–20 system, such as FC3, C3, etc.) was used to collect the brain electrical signals with a sampling rate of 256 Hz and a band‐pass filter ranging from 0.5 to 30 Hz. The reference electrode was placed on the right earlobe, and the ground electrode was located at FPz. The μ and *β* rhythms related to MI were monitored in real time.	Closed loop: The MI‐contingent group only triggers functional electrical stimulation (FES, 50 Hz, 300 µs pulses) to stimulate the wrist extensor muscles when the system detects the correct MI and synchronizes the virtual avatar's visual feedback. Open loop: The MI‐independent group triggers FES and visual feedback at regular intervals regardless of whether the MI is correct.	Offline stage: Utilize the Common Spatial Pattern (CSP) method to optimize spatial filtering and apply LDA for classification of MI signals. Online stage: Real‐time analysis of EEG signals. Trigger feedback when MI‐related features are detected.	The BCI group receiving MI‐related feedback showed significant improvements in wrist extensor strength (MRC‐WE) and wrist extension range of motion (AROM‐WE) compared to the BCI group receiving MI‐irrelevant feedback. The BCI group receiving MI‐related feedback exhibited increased functional connectivity in the frontal premotor area of the affected hemisphere, which was correlated with MRC‐WE and Fugl–Meyer assessment‐distal (FMA‐distal) scores. Functional connectivity improved in both hemispheres.	[203]
2023	Rubin et al.	Quadriplegia due to SCI, brainstem stroke, or motor neuron disease.	Fourteen adults aged 18–75 years with quadriplegia due to SCI, brainstem stroke, or motor neuron disease	One or two Utah MEAs (96 channels, 4 × 4 mm platform) were implanted into the motor cortex of the dominant hemisphere and connected percutaneously through the skull base. Single neuron discharges and LFPs were recorded in real time. The sampling rate was not specified, and a band‐pass filter with a range of 0.5–30 Hz was used.	Closed loop: The screen displays visual feedback of the virtual arm movements, which is synchronized with the brain's electrical code results. Some participants received FES stimulation of the limb muscles. The system in real time decodes and updates the feedback to enhance neural plasticity.	Offline stage: Train the motion intention classification model using CSP and LDA. Online stage: Real‐time decode the electroencephalogram signals and trigger FES or virtual avatar feedback.	The safety record of the BrainGate system was comparable to that of other chronic implantable medical devices. No serious adverse events (SAEs) requiring device removal, resulting in death, or inducing permanent disability occurred during the 1‐year post‐implantation evaluation. The most common adverse event was skin irritation, mainly related to excessive cleansing of the skin around the surgical incision or implantation site. Two participants developed seizures after surgery, but they did not have further seizures and continued to participate in the clinical trial. There was a small amount of adherent tissue on the arrays more than 5 years after implantation.	[204]
2019	Caria et al.	Stroke	30 patients with severe chronic stroke	Using an 8‐channel EEG cap to collect brain electrical signals related to motor imagination (at C3, C4, and other sites), with a sampling rate of 256 Hz, the phenomenon of μ rhythm suppression was monitored in real time.	Closed loop: In the VR scene, the virtual arm moves according to the MI decoding result; only when the MI decoding is correct, the FES stimulates the target muscle group (current intensity 10–30 mA). The system drives feedback through real‐time electrocoding of the brain, and the patient needs to actively adjust the MI to maintain the interaction.	Offline phase: Use the Public Space Pattern (CSP) and SVM to train the MI classification model. Online phase: Real‐time decode the MI intention, with the delay of triggering feedback <0.5 s.	Brain–machine interface (BMI) therapy can induce remodeling of motor networks in patients with severe chronic stroke. By enhancing the activity of the affected motor cortex and improving the proprioceptive function of the affected hand, BMI treatment can promote remodeling of the affected and healthy sensorimotor cortex and motor circuits associated with afferent and efferent connections, respectively, thereby partially restoring motor system function.	[205]
2020	Palanker et al.	Atrophic age‐related macular degeneration (AMD)	Five patients with age‐related macular degeneration and macular atrophy. The atrophic area was at least 3 optic disc diameters, and the best corrected vision was 20/400 to 20/1000.	A 2 mm wide and 30 µm thick chip, containing 378 photovoltaic pixels with a diameter of 100 µm, was implanted in the subretinal atrophy area. Near‐infrared light (880 nm) was projected from the video glasses. The pixels directly converted the light into local currents, stimulating the adjacent bipolar cells and other inner retinal neurons.	Simple stimulation: The stimulation parameters (light intensity, pulse frequency) are preset by an external device, and there is no real‐time neural signal feedback for regulation.	The external camera captures images, which are processed and then projected onto the retina via video glasses using NIR light. The pixel array generates differences in current intensity based on the image's grayscale. Brainwave signal decoding relies on preset light stimulation patterns.	All five patients were able to perceive white or yellow visual patterns in the previously dark areas, and three of them had ideally positioned implants, with a visual clarity of 20/460–20/550, while the other off‐center patient had a visual clarity of 20/800. In all patients, residual natural vision did not decline after implantation. This study shows that photovoltaic retinal implant for macular atrophy (PRIMA) implants can safely and effectively restore central vision in patients with atrophic AMD without affecting peripheral vision.	[206]
2024	Muqit et al.	AMD	Five patients with atrophic AMD and no macular light sensation in the affected eye (logarithm of the minimum angle of resolution [logMAR] 1.3–1.7, 20/400–20/1000).	Implant a 2 mm wide and 30 µm thick photovoltaic microchip, containing 378 pixels with a diameter of 100 µm, beneath the atrophic area of the retina. Near‐infrared light (880 nm) is projected through augmented reality (AR) glasses. The pixels directly convert the light into local currents, stimulating inner retinal neurons such as bipolar cells.	Simple stimulation: The stimulation parameters (light intensity, pulse frequency) are preset by an external device, without a closed‐loop interaction mechanism.	The external camera captures images, which are processed by the pocket processor and then projected onto the retina through AR glasses. The pixels generate current intensity differences based on the image's grayscale. There is no decoding of brain signals, relying on preset light stimulation patterns. There is no real‐time feedback of neural signals.	Implantation of the PRIMA subretinal implant was feasible and well tolerated, with no reduction in natural peripheral vision over 48 months. The central vision prosthesis provided by photovoltaic nerve stimulation enabled patients to reliably recognize letters and letter sequences, and using the zoom function improved vision by up to 8 lines of ETDRS letters.	[207]

## 5 Advances in Materials Science for Improved BCI Electrodes

Most electrodes for invasive BCIs are made of metals such as tungsten, which have high conductance but poor histocompatibility [208], and strain and stress around the implant site can eventually result in severe inflammation and brain tissue damage [7, 209, 210]. The inflammation induced by electrode movement and tissue damage, in turn, leads to the proliferation of reactive glial cells, forming glial scars, while other immune cells and fibroblasts form inflammatory deposits encapsulating the electrodes. When coupled with the adhesion of proteins and other components on the surface, this process increases electrode impedance. Inflammatory cells also produce and release cytotoxic reactive oxygen species as well as degradative enzymes and acids, resulting in the degradation of electrode materials. The result is gradually increasing impedance, a decrease in SNR, and ultimately electrode failure [211, 212, 213]. In cases of electrodes designed to also deliver drugs, these processes may stimulate excessive release.

These problems have spurred intensive research on alternative electrode materials, such as carbon fiber, titanium, polymers, nanomaterials, and even biomaterials [214, 215, 216]. In this section, we will describe advances in electrode materials for BCI.

### 5.1 Innovative Electrode Materials

Platinum electrodes are now widely used for long‐term nerve stimulation and recording applications due to the excellent corrosion resistance, good biocompatibility, and non‐allergenic properties of this metal. In addition, platinum is amenable to microfabrication techniques for the construction of high‐density, high‐precision electrode arrays [217]. Titanium compounds, such as Ti_3_C_2_ MXene, are also useful electrode materials due to low impedance, high SNR, and good biocompatibility [218]. For stimulating electrodes, iridium oxide has a high charge injection capacity and low impedance [219], while the transparency of indium tin oxide electrodes is ideal for optical imaging and optogenetic studies [220]. In addition, silicon has good biocompatibility. Moreover, bulk silicon nanomembranes were found to dissolve in phosphate‐buffered saline at 37°C over 12 days, indicating good absorption properties for degradable electrodes. Such degradable electrodes can be fabricated by attaching silicon nanomembranes to a bioresorbable polylactic acid‐glycolic acid copolymer substrate. These electrodes degrade automatically after a period of implantation for transient usage, thereby eliminating the risks involved in secondary surgical removal [217]. The unique properties of many metal‐based nanomaterials also appear ideal for BCI electrodes. For example, magnetoelectric nanodisks (MENDs) can convert magnetic fields into electrical signals, and these metal nanomaterials can be injected into the brain and trigger calcium signaling responses in neurons under magnetic field stimulation. Such electrodes could theoretically replace genetic approaches such as optogenetics, where light‐activated ionic channels are expressed by target neurons for control of membrane excitability. The use of MENDs could enable wireless regulation of neural activity, providing a more minimally invasive alternative to DBS [221]. Moreover, room‐temperature liquid metals are suitable for long‐term neurostimulation applications owing to their high electrical conductivity and excellent adaptability [222]. For instance, gallium‐based alloys such as eutectic gallium‐indium (EGaIn) exhibit favorable electrical conductivity and mechanical properties. Research indicates that EGaIn can be fabricated into flexible neural electrode arrays, which adapt to the dynamic changes of brain tissue while maintaining good biocompatibility and stability during long‐term implantation [223]. Additionally, EGaIn can be utilized to create neural conduits for repairing peripheral nerve injuries [224].

### 5.2 Carbon Nanomaterials for BCI

Carbon nanomaterials have also emerged as promising candidates for BCI electrode fabrication due to their unique and highly modifiable physical and chemical properties. Unlike many other nanoparticle materials, carbon is inherently biocompatible and non‐toxic, and carbon‐based nanomaterials are lightweight, porous, flexible, electrically conductive, and stable, ideal for implantable neural interfaces and neural tissue engineering applications [146]. Carbon nanomaterials can greatly improve the efficiency of neural signal recording and brain stimulation by reducing electrode impedance and noise [225, 226]. Carbon nanomaterials can also be fabricated as flexible electrode arrays for large‐area brain mapping and chronic in vivo recording, and the structural flexibility of electrodes coated with carbon nanoparticles provides a good mechanical match to surrounding tissues, which can reduce nerve tissue damage and inflammatory responses [227, 228]. Carbon nanomaterials can be divided into three types according to dimension: zero‐dimensional, such as fullerenes and nanodiamonds; one‐dimensional, such as nanotubes; and two‐dimensional, such as graphene and MXenes. Carbon nanotubes (CNTs) have particularly good electrical conductivity and flexibility for use in neural interface systems. Similarly, MXene has a rich functional group and good electrical conductivity for use at bioelectronic interfaces, while graphene has excellent electron mobility and electrocatalytic activity ideal for biosensing electrodes. Other advantages of graphene include transparency, conductivity, biocompatibility, and mechanical flexibility, ideal for the future development of micro‐ECoG technology and various long‐term applications. The transparency of graphene is also advantageous for optogenetic BCIs [229].

Although CNTsand MXenes exhibit favorable initial mechanical properties, prolonged exposure to the dynamic cerebral environment—characterized by mechanical stresses such as compression, tension, and shear forces in brain tissue—may lead to material degradation. The long‐term utility of CNTs is constrained by surface hydrophobicity, residual metal catalysts, and interference with ion channels, while MXene's stability requires further investigation [230]. To address these challenges, researchers have developed MXene/CNT heterostructures [231]. These leverage ester bond linkages to expand interlayer spacing, facilitating ion migration and storage, thereby enhancing mechanical deformability and cyclic stability. Alternatively, cationic amino‐functionalized multi‐walled CNTs serve as interlayer spacers, mitigating MXene nanosheet restacking while promoting charge transfer. This configuration improves electric double‐layer capacitance and mechanical stability by preventing interfacial water layer formation [232].

For CNT hydrophobicity, surface functionalization with hydrophilic groups (e.g., carboxyl, hydroxyl, amino, or polyethylene glycol) enhances biocompatibility and stability [233]. Incorporation of bioactive molecules such as neurotransmitter receptors or cell adhesion factors further strengthens neural tissue integration [234]. Composite systems integrating carbon nanomaterials with elastic polymers (e.g., polydimethylsiloxane [PDMS]) significantly improve mechanical elasticity and electrochemical stability [235, 236]. Additionally, CsPbBr_3_ quantum dots coupled with MXene form hybrid structures where quantum dots boost photocurrent generation, excitatory postsynaptic currents, and signal linearity, while MXene nanosheets act as mechanical scaffolds. This synergy achieves high optoelectronic efficiency and mechanical robustness [237]. Collectively, these strategies overcome material limitations in dynamic biological environments, advancing their applicability in neural interfaces. Additionally, emerging materials like bacterial cellulose [215] and conductive hydrogel [238] show promise in balancing biocompatibility and long‐term stability, representing frontier directions in electrode design.

### 5.3 Composite Materials for BCI Applications

Composite materials that combine polymer materials, metal materials, and biomaterials, such as conductive hydrogels, are also evolving [235]. Conductive polymers, such as PEDOT:PSS, are considered ideal for neural electrodes due to their low impedance, high charge storage capacity, and favorable mechanical properties [239]. Hydrogel materials also serve as excellent interfacial materials for connecting flexible electronic devices with biological tissues, owing to their superior stretchability and biocompatibility [222]. A flexible Cu‐TiO_2_‐CNT‐PDMS composite fabricated by adding copper sulfate crystals, titanium dioxide nanoparticles, and CNTs to a PDMS matrix demonstrated high flexibility, adhesion, and biocompatibility as well as antimicrobial properties [56]. These materials have shown considerable promise for maintaining long‐term stability at neural interfaces by preventing scarring and inflammatory tissue damage caused by electrode implantation [226]. Moreover, these electrodes can be designed for the on‐demand delivery of anti‐inflammatory drugs and neurotrophic factors. Zhu et al. coated nichrome electrodes and CNT fiber electrodes with a composite consisting of sulfonated quartz nanoparticles loaded with melatonin (MT) in poly(3,4‐ethylenedioxythiophene) (PEDOT) by electrochemical deposition (PEDOT/SNP‐MT‐coated electrodes) to achieve controlled release of MT with electrical stimulation and found a significant reduction in the number of astrocytes at the implantation site [240]. This study highlights the potential of novel composites for improving the stability and prolonging the lifetime of implantable electrodes. Similarly, polyaniline–gelatin–sodium alginate conductive hydrogel coatings and dexamethasone‐doped PEDOT coatings have also been demonstrated to reduce electrode‐site inflammation and maintain stability for long‐term use [238, 241].

In the field of BCI technology, the introduction of intelligent and biodegradable materials plays a significant role in reducing the risk of secondary surgeries and enhancing the long‐term performance of implants. Stimuli‐responsive polymers such as pH‐ and temperature‐sensitive materials can automatically adjust their structure and function in response to changes in the in vivo environment, enabling self‐adaptation and degradation. pH‐responsive polymers typically respond to changes in the body's pH by altering their surface charge or chain structure. For example, in acidic environments such as tumor microenvironments or inflamed tissues, the polymer chains undergo protonation reactions, leading to chain cleavage or structural changes [242]. Additionally, the degradation pH of these polymers can be precisely controlled by adjusting the ratio of functional groups within the polymer. For instance, degradation can occur rapidly at pH 5.0 but remains stable at physiological pH 7.3 [243]. Temperature‐responsive polymers, on the other hand, regulate their functionality through thermoresponsive phase transitions. A well‐known example is poly(N‐isopropylacrylamide), which undergoes a conformational transition from extended to collapsed states near body temperature (approximately 32°C), thereby modulating its solubility and permeability [244]. This temperature‐dependent behavior is exploited in the design of self‐healing or deformable implantable devices to adapt to in vivo conditions [245]. Nanoscale silicon films achieve controlled degradation through surface chemical modification and structural design. For example, the introduction of thiol groups or the regulation of nanopore density can modulate the degradation rate of silicon nanomaterials [246]. The degradation process of nanosilicon typically involves hydration, hydrolysis, and ion exchange, all of which are influenced by particle size, surface functionalization, and environmental pH [247]. By precisely controlling these parameters, nanosilicon films can achieve controlled degradation under specific physiological conditions, thereby reducing the need for secondary surgeries [245].

In recent years, nanozyme‐based electrode optimization has emerged as a highly promising strategy, offering innovative pathways to enhance the overall performance of BCI electrodes. By mimicking the catalytic activity of natural enzymes, nanozymes exert unique effects at the electrode interface. The core advantage of this optimization strategy lies in the ability of nanozymes to significantly improve the electrochemical properties (e.g., reducing interfacial impedance, enhancing charge transfer efficiency) while simultaneously enhancing biocompatibility (e.g., mitigating inflammatory responses, suppressing glial scar formation [248]). The underlying mechanism involves utilizing their enzyme‐like catalytic activity to modulate the microenvironment at the electrode‐tissue interface, thereby synergistically optimizing both electrical signal transmission and biological tissue responses. Research has confirmed that functionalized coatings based on nanozymes, such as platinum nanoparticles, effectively maintain the stability and biocompatibility of chronically implanted electrodes [249]. Such strategies represent a significant breakthrough direction in electrode design, providing a novel paradigm to resolve the inherent trade‐off between electrical performance and biocompatibility inherent in traditional electrode materials. Furthermore, advances in the design of novel nanozymes, including metal‐organic frameworks, are expected to further advance the development of neural interfaces with a high SNR and superior biocompatibility upon their integration into BCI electrodes [250].

In addition to selecting highly biocompatible materials for the manufacture of electrodes, such as platinum, iridium, titanium, and conductive polymers, various research groups are exploring new electrode materials with modifications such as surface coatings, peptide deposition, and laser or plasma modification mechanisms (e.g., hierarchical electrodes [251]) to improve electrochemical performance and reduce tissue reactions [252, 253]. Several studies have also reported that electrodes coated with platelet plasma and mesenchymal stem cells can promote the regeneration of neural tissue and reduce the inflammatory response [254, 255, 256]. Also, there is currently an intensive effort to develop encapsulation materials that resist degradation. One study found that the encapsulation material surrounding a UTAH electrode array implanted in the human cerebral cortex gradually degraded after implantation, which led to a decrease in signal quality [257]. Therefore, the development of new protective materials (e.g., polymers) is essential to prevent material degradation and maintain electrode performance for long‐term BCI applications. For example, depositing a silica film on the surface of high‐temperature metal electrodes can effectively enhance electrode stability and extend electrode longevity [234].

Furthermore, synthesis methods for novel neural electrode materials, such as wet spinning, laser patterning, and 3D printing, help optimize the mechanical properties, electrochemical performance, and biocompatibility of electrodes. This meets the requirements of neural interfaces for long‐term stability and high sensitivity. Wet spinning is utilized to fabricate freestanding electrodes with controllable pore structures, enabling precise control over fiber diameter and surface morphology [258]. This enhances electrochemical activity and mechanical strength and is widely applied in producing graphene oxide fibers and CNT fibers [259]. Through laser patterning and 3D printing, micro/nano‐scale processing and microstructural optimization of electrodes can be achieved, facilitating the fabrication of polymer coatings. These advancements improve insertion success rates in brain tissue and long‐term stability [260, 261].

**FIGURE 5:**
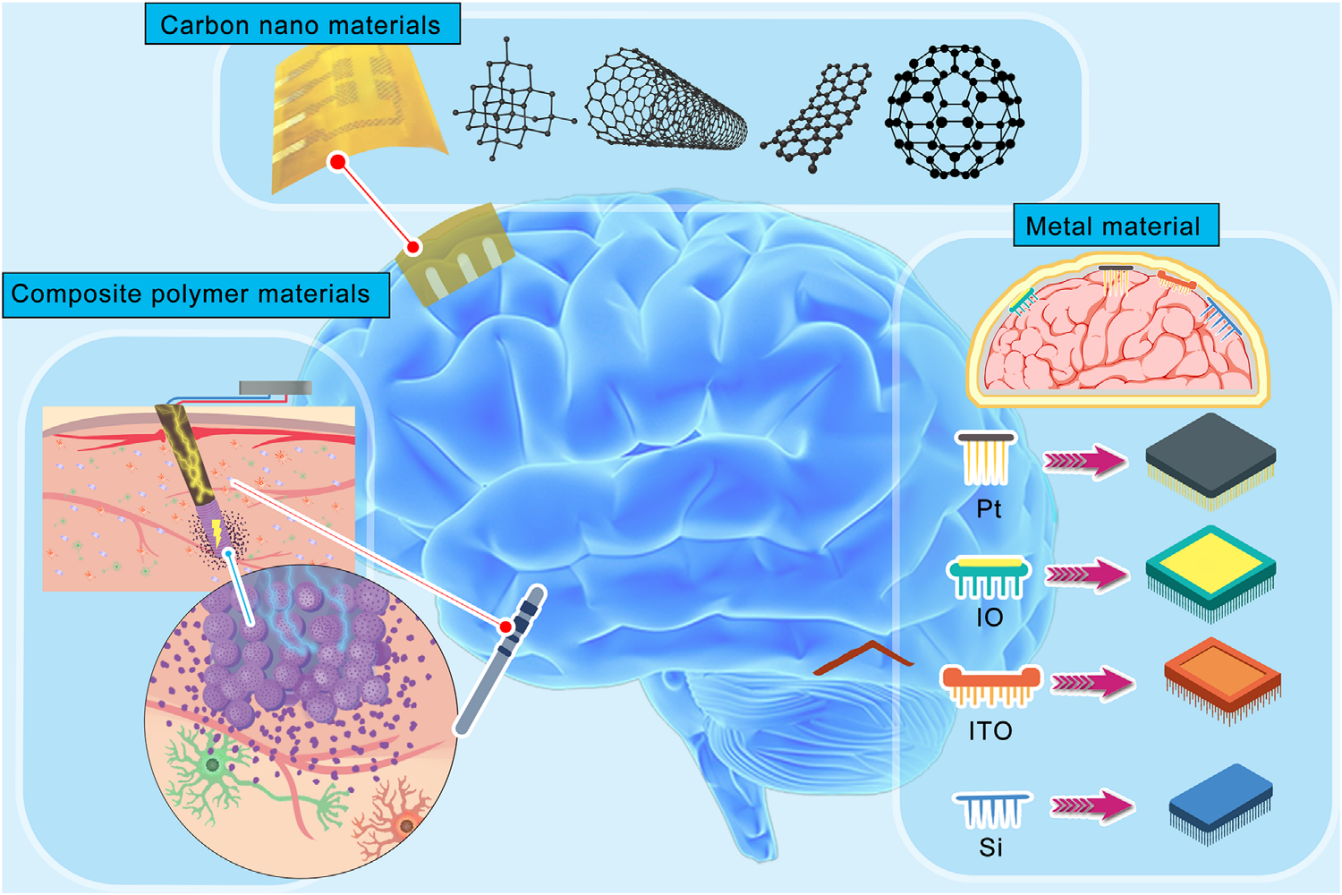
Advances in BCI electrode materials. A variety of materials are used for BCI electrode fabrication, each with individual advantages and disadvantages. Newer electrode metal materials included platinum (Pt), iridium oxide (IO), indium tin oxide (ITO), and silicon (Si). PEDOT/SNP‐MT is shown as an example of a composite polymer material used to coat carbon nanotube (CNT) electrodes in the cerebral cortex for combined stimulation and drug delivery. PEDOT (poly(3, 4‐ethylenedioxthiophene)) is a flexible hydrogel polymer with quartz nanoparticles (SNPs) attached. These porous electrodes can be loaded with melatonin (MT), which is released upon stimulation into the surrounding tissues. The carbon fiber materials section illustrates three dimensions of these materials, carbon nanotubes, graphene, and Mxene. Another image shows a flexible carbon fiber electrode used for implantation.

Some of these new materials with promising applications in BCI systems are illustrated in Figure 5. In addition, Table 4 compares the properties of several different materials.

**TABLE 4:** Comparison of key performance characteristics of brain–computer interface electrode materials.

Material system	Electrical property	Charge storage capacity (CSC)/charge injection limit (CIL)	Mechanical properties	Glial scarring	Special attributes	Ref
PEDOT:PSS/Au	Impedance: 121 MΩ.µm^−2^(Electrode surface area: 61 575 µm^2^)	CIL: 2.5 mC cm^−2^	/	/	High ion‐electron coupling capability	[262]
PEDOT/SNP‐MT	Impedance: Ni‐Cr alloy electrode: Before coating: 1356.2 kΩ After coating: 192.8 kΩ Reduction rate: 85.7% CNT electrode: Before coating: 125.2 kΩ After coating: 520.5 kΩ Increase rate: 316.4%	CSC: Ni‐Cr alloy electrode: Before coating: 3.2 pC After coating: 44.2 pC Increase rate: 1281.3% CNT electrode: Before coating: 18.7 pC After coating: 88.8 pC Increase rate: 475.5%	/	Ni‐Cr alloy electrode: Reduced by 60.11%. CNT electrode: Reduced by 25.69%.	Controllable release of melatonin	[240]
PEDOT	Electrical conductance:1200 S·cm^−1^	/	Young's modulus: 2.6 ± 1.4 GPa	/	Limited transparency	[263]
Cu‐TiO_2_‐CNT@PDMS	The impedance of the scalp contact area is less than 5kΩ; the impedance of the scalp contact area with hair is less than 20 kΩ.	/	The compressive strength reached 15 MPa, the strain exceeded 70%, and it remained undamaged.	/	Good biocompatibility	[56]
Graphene	Electrical conductance: 1.0 × 105 S/m	/	Young's modulus: 950–1000 GPa	/	Nontoxic Rare allergenic	[264]
CNT fiber	Impedance: 20.44 MΩ.µm^2^	CIL: 6.52 mC.cm^−2^	/	/	MRI artifacts were reduced by 80%	[265]

## 6 Endovascular BCI, Optogenetics BCI, and Other Emerging Directions

As discussed, traditional invasive electrode placement methods can lead to a range of complications such as hematoma, infection, and blood–brain barrier disruption. Further, implantable electrodes are not suitable for signal acquisition across large areas or multiple recording sites. However, non‐invasive scalp electrode arrays have limited measurement depth, spatial precision, and sensitivity [266]. To circumvent these limitations, Mitchell and colleagues have proposed a new endovascular pathway for BCI electrode implantation [267]. This endovascular approach is significantly less invasive than open surgery, thus reducing postoperative recovery time and the risk of infection. In addition, many important neuromodulation targets in the central and peripheral nervous systems are located in the vicinity of vascular structures, so delivery of electrical stimulation is still possible [268]. Compared to traditional invasive or non‐invasive BCI electrodes, intravascular BCI electrodes can also record from or stimulate deeper brain regions. Further, the intravascular environment is relatively stable, reducing the risk of electrode displacement and improving overall electrode reliability and longevity for signal recording. Intravascular electrodes can also be used to stimulate both brain tissue and peripheral nerves as an alternative to DBS, TMS, and FES [269]. Also, by incorporating a closed‐loop stimulation system, intravascular BCI can dynamically adjust stimulation parameters based on real‐time monitoring of brain activity, optimizing treatment outcomes [270].

It is expected that signal measurements and stimulation will be further improved by the development of smaller and more flexible miniature electrodes and by the optimization of the design of intravascular electrodes. For example, ultra‐miniature electrodes fabricated with flexible electronic materials and nanotechnology can better adapt to the intravascular environment and reduce damage to blood vessel walls, improving the fidelity and duration of signal acquisition. In addition, closed‐loop control systems combined with AI algorithms could provide more accurate individualized neuromodulation for the treatment of neurological diseases. In conclusion, interventional BCI, as an innovative technology path, is expected to become an important tool for the treatment of neurological diseases, providing patients with safer and more effective treatment options.

Brain stimulation techniques have also improved owing to developments in materials science, greater knowledge of brain networks, and novel stimulation modalities. These modalities can be divided into three main categories: electromagnetic stimulation [271], ultrasound stimulation [272, 273], and photostimulation [274]. The most common magnetic stimulation technique is currently TMS [275]. Electromagnetic stimulation modalities such as conventional DBS, rTMS, TBS, tDCS, and TMS are widely used in BCI systems but have low spatial resolution [276]. In contrast, ultrasound stimulation and photostimulation can be highly precise. Ultrasound technology uses sound waves to act on mechanically sensitive voltage‐gated ion channels, opening the channels through mechanical deformation to regulate neuronal excitability and circuit activity. The two major forms of ultrasound stimulation are transcranial‐focused ultrasound and transcranial unfocused ultrasound [277]. Compared to electromagnetic stimulation, ultrasound stimulation has higher spatial resolution and penetration depth, but its application in BCI is still in the exploratory stage. Photostimulation techniques, particularly optogenetics, enable precise regulation of neuronal and neural circuit excitability, including deep neurons and circuits, because photosensitive channel expression is restricted to target neurons [278]. Optogenetics BCI has made rapid progress in recent years and has been widely used in laboratory research. Compared to traditional BCI technology, optogenetics BCI has shown other unique advantages. First, by adjusting the wavelength, frequency, and power of light stimulation, combined with the localized expression of photosensitive proteins, optogenetics can achieve precise regulation of specific neuronal populations at specific time points and avoid interference by non‐target neurons [279, 280]. Second optogenetics BCIs support bidirectional regulation of neurons by selectively activating or inhibiting neuronal activity [281, 282]. In addition, optogenetics can enhance neuronal plasticity and provide new possibilities for neurorehabilitation [283, 284]. In experimental studies, optogenetics BCI has been successfully applied to the precise regulation of a variety of brain regions. For example, the cortex was stimulated by optogenetics to achieve significant inhibition of pain perception in rats and precise regulation of tactile perception in mice [283, 284, 285, 286, 287]. The optogenetic approach has also provided a new experimental strategy for elucidating the neural mechanisms of depression and evaluating potential treatments [288]. These research results not only support the effectiveness of optogenetics BCI for neuromodulation but also lay the foundation for its application in a wider range of fields.

Optogenetics BCI has also shown potential in the fields of neural circuit function analysis, limb motor function rehabilitation, and neurological disease treatment. For example, by incorporating virtual sensory feedback technology, optogenetics BCIs are expected to achieve high‐precision decoding of neural inputs, thereby providing prosthesis users with a more realistic limb perception experience. In addition, with the rapid development of closed‐loop BCI technology, it has become possible to combine closed‐loop regulation with optogenetics BCI. By monitoring neural activity in real‐time and dynamically adjusting photostimulation parameters (e.g., light intensity and frequency), closed‐loop optogenetics BCI enables more efficient, precise, and personalized neuromodulation. This technological integration not only improves the spatiotemporal resolution and control accuracy of optogenetics but also provides an innovative technical path for the individualized treatment of neurological diseases.

The integration of optogenetic techniques into BCIs presents both innovative potential and significant technical and ethical challenges. While optogenetic control offers unprecedented opportunities for neural decoding due to its precision, the requirement for genetic modification introduces practical barriers, including limitations in light penetration depth and risks associated with viral delivery vectors, such as concerns over non‐targeted expression and immune responses, alongside the potential for brain tissue damage caused by fiber optic implantation [7]. To overcome these issues, several advancements are being pursued. Development of wireless optoelectronic BCIs demonstrates improved tissue compatibility and reduced surgical invasiveness, while micro‐optrodes enhance spatial resolution and minimize tissue injury [7]. Concurrently, non‐viral gene delivery systems, including electroporation [289] and nanoparticles, are being optimized for targeted expression in specific neuronal populations, mitigating risks of immune reactions and chronic inflammation [290]. Furthermore, enhanced optogenetic tools with faster activation kinetics, such as channelrhodopsin variants, reduce the need for high‐intensity light irradiation, thereby increasing safety [291]. To address light penetration limitations, upconversion nanoparticles [292] enable excitation in deep tissue using low‐energy near‐infrared light, reducing phototoxicity. Additionally, multimodal integration employing non‐invasive techniques like fNIRS or fMRI allows for monitoring and feedback, lessening reliance on deep tissue stimulation [7]. Moreover, ultrasonic genetics [277], a non‐invasive neuromodulation method capable of activating mechanosensitive ion channels without penetrating the skull, presents an alternative, although its translational potential remains to be fully validated. Ethically, while technological progress is crucial, the clinical translation of optogenetic BCIs necessitates robust frameworks. Clinical translation requires prioritizing biosafety in preclinical studies and employing stringent patient selection criteria in clinical trials, for instance, focusing on end‐stage diseases where benefits clearly outweigh risks. Ultimately, personalized treatment planning and rigorous informed consent protocols are essential to balance therapeutic benefits against potential risks. Furthermore, multidisciplinary collaboration among neuroscientists, engineers, and ethicists is imperative to ensure responsible innovation and address societal implications [293]. Future research should prioritize long‐term biocompatibility and scalable manufacturing to accelerate the clinical viability of these technologies.

The development of carbon nanomaterials is among the most valuable recent technical advances for BCI systems. First, carbon nanomaterials have excellent electron transport properties and surface oxide adsorption capacity [294], enabling them to detect a variety of neurotransmitters and biochemical markers with high sensitivity, including dopamine, ascorbic acid, serotonin, L‐lactic acid, nitric oxide, and inflammatory factors [295, 296, 297, 298]. Changes in the concentration of these neurotransmitters and biochemical molecules are closely related to the pathological mechanisms underlying a variety of neuropsychiatric disorders, such as PD, AD, MDD, schizophrenia, and drug addiction [299, 300, 301]. Therefore, carbon fiber electrodes can not only monitor the dynamic changes of neurotransmitters in real time but also assess the progression of neurological diseases, providing an important basis for early diagnosis and precise treatment [302, 303]. In addition, carbon nanomaterials are highly suitable as drug delivery platforms due to their rich lumen structure and good biocompatibility. Indeed, carbon nanomaterials have been successfully used for the targeted release of a variety of drugs, including the anti‐inflammatory dexamethasone, glutamate AMPA/kainate receptor antagonist DNQX, the antitumor agent doxorubicin, sodium fluorescein, and insulin‐like growth factor‐1 [304, 305, 306, 307]. This drug delivery capability enables carbon fiber electrodes to not only detect neural activity but also modulate activity through local drug release, a capacity with potential treatment applications for a myriad of neuropsychiatric disorders. For example, in the treatment of PD, carbon fiber electrodes can achieve dynamic regulation of the disease by monitoring dopamine levels in real time and releasing therapeutic drugs in addition to exciting relevant neural pathways involved in motor control. With further optimization of carbon nanomaterial preparation technology and improvements in BCI algorithms, carbon electrode BCI is expected to become a routine strategy for the diagnosis and treatment of neuropsychiatric disorders.

## 7 Selection of Animal Models and Pathways for Clinical Translation

In BCI research, the selection and optimization of animal models are critical for achieving long‐term (>6 months) implantation studies. The choice of animal models must be based on their alignment with the pathophysiology, genetic characteristics, and behavioral phenotypes of the target diseases. For example, in movement disorders, genetically modified mice or rats (e.g., SNCA mutant mice, Huntington's disease mouse models) can simulate the pathological features of PD or Huntington's chorea [308, 309, 310]. Similarly, models of cerebral ischemia, traumatic brain injury, and SCI include transient middle cerebral artery occlusion models in mice and bilateral cerebral ischemia models in rats [311]. However, rodents often exhibit signal degradation and unstable tissue responses after long‐term implantation, such as meningeal encapsulation and neuronal damage, whereas non‐human primates, such as macaques, are better suited for long‐term signal recording and chronic implantation due to their anatomical similarity to the human brain [312].

In psychiatric disorders, the selection of animal models must consider the similarity between their behavioral phenotypes and those of human diseases. For instance, transgenic mice with specific gene mutations (e.g., DISC1, COMT) can be used to model early symptoms of schizophrenia [313]. However, these models often fail to fully replicate the complex emotional and cognitive impairments observed in human psychiatric disorders [312]. In neurodegenerative diseases such as AD, animal models include APP/PS1 mice and TgCRND8 AD mice, while PD models are typically induced by 6‐hydroxydopamine (6‐OHDA) injections [311]. These models are valuable for studying the pathophysiology and therapeutic strategies of neurodegenerative diseases, but their limitations in capturing the complexity of multi‐system involvement are evident [314].

In the context of BCI applications, the selection of animal models must balance the complexity of the disease, signal stability, and the controllability of tissue responses. Long‐term implantation studies require not only the stability of signals but also the integration of behavioral and neuropathological assessments to comprehensively evaluate the suitability of the model. Behavioral assessments (e.g., open‐field tests, social interaction tests) can quantify behavioral abnormalities in the model, thereby assessing its similarity to human diseases. Neuropathological assessments (e.g., tissue section analysis, immunohistochemistry) help evaluate the effects of chronic implantation on neural tissue, such as neuronal degeneration, axonal injury, and myelination [315]. The combination of these assessments enhances the predictive power of the model and provides critical insights for optimizing the design and materials of implantable devices.

However, the limitations of animal models in clinical translation are significant. Species differences can lead to inconsistencies in behavioral phenotypes and neural mechanisms. For example, although rodent models can simulate certain early symptoms of psychiatric disorders like schizophrenia, they often fail to fully replicate the complex emotional and cognitive impairments seen in humans [316]. Additionally, many therapeutic strategies that show promise in animal models have failed in clinical trials, highlighting the need for improved predictive accuracy and generalizability [317]. Future research should focus on refining the selection criteria for animal models, integrating long‐term behavioral and neuropathological assessments, and developing advanced implantable devices and materials. The adoption of multimodal analytical approaches, such as automated behavioral monitoring, in vivo imaging, and epigenetic analysis, will further enhance the translational potential of animal models and accelerate the development of BCI technologies for real‐world applications [318].

The BCI technology faces numerous challenges during its clinical application process, including technical maturity, regulatory approval, patient acceptance, and long‐term efficacy assessment. Studies have shown that the clinical transformation of BCI requires multidisciplinary collaboration, involving the deep integration of fields such as neuroscience, engineering, AI, and clinical medicine [319]. For instance, the application of non‐invasive BCI technology in the fields of assistive and alternative communication has shown promising results. However, its large‐scale clinical application still requires addressing issues such as the stability of the equipment, user training, and personalized adaptation [320]. Therefore, future research should focus on how to promote the clinical application of BCI technology by optimizing algorithms, enhancing signal processing capabilities, and developing user‐friendly interfaces [321].

From the perspective of technological transformation, the clinical application of BCI typically goes through the following key stages: basic research and prototype development, preclinical research and animal experiments, clinical trials and ethical review, regulatory approval and product standardization, as well as large‐scale clinical application and long‐term follow‐up. During the basic research stage, researchers need to clearly define the target population and clinical needs, such as restoring motor function in paralyzed patients or achieving early warning in epilepsy patients. Subsequently, the prototype device needs to undergo animal experiments to verify its safety and effectiveness and then enter the clinical trial stage to assess its performance in real environments and potential side effects [319, 322, 323].

During the clinical transformation process, regulatory and ethical issues cannot be ignored either. For instance, the Food and Drug Administration in the United States and the CE certification system in Europe have relatively strict approval procedures for BCI devices, requiring the provision of sufficient clinical evidence and long‐term safety data. Furthermore, patient privacy protection and data security are also important ethical issues that must be taken into consideration in the clinical application of BCI. Studies have shown that establishing interdisciplinary cooperation mechanisms (such as neuroengineers, clinicians, ethicists, and policymakers) is crucial for the smooth transformation of BCI technology [319, 323, 324].

The clinical application of BCI not only depends on technological advancements but also requires the establishment of a complete medical service system and a user support system. For instance, users of BCI devices may require long‐term rehabilitation training and psychological support to facilitate their sustained use in daily life. Therefore, beyond technological optimization, future study should also focus on establishing an ecosystem that supports the clinical application of BCI, covering multiple aspects such as policy, healthcare, education, and industry, and achieving collaborative efforts in all these areas [319, 323, 324].

## 8 Discussion

The BCI‐based treatment is currently transitioning from unimodal to multimodal systems driven by recent advances in neuroengineering. These multimodal BCI systems feature not only multiple neural signal acquisition technologies, such as EEG, fNIRS, fMRI, and MEG [325], but also therapeutic methods, such as DBS, FES, and XR [84, 121, 326]. Multimodal BCI obviates many limitations of monotherapy and has unique technical advantages. For example, combining fNIRS, fMRI, and EEG to monitor neural activity during seizures provides a comprehensive description of the pathological state with high spatiotemporal resolution. Similarly, by combining BCI with DBS and FES, the system can achieve both central nervous system regulation and peripheral limb activation with high spatiotemporal precision to achieve a “central‐peripheral” two‐way intervention model. Indeed, the clinical value of multimodal BCI has been validated by several studies [76, 327, 328]. For instance, an RCT reported that patients with post‐stroke motor dysfunction receiving cognitive training, balance training, and vibration therapy using a multimodal BCI + exoskeleton system achieved better functional recovery than those receiving unimodal BCI + exoskeleton training [329].

Generally, multimodal BCI systems form a complete “perception‐decision‐execution‐optimization” closed‐loop by integrating signal acquisition, decoding algorithms, actuators, and feedback systems. These systems not only obviate the limited spatiotemporal resolution, signal specificity, and regulation dimensions of single‐modality BCIs but also improve the accuracy of motion intention decoding. In addition, feedback combined with various adaptive algorithms (e.g., machine learning tools) can optimize individualized parameters for improved efficacy and user control. These innovations provide a new technical paradigm for the precision rehabilitation of disorders and also lay an important foundation for future clinical translation.

Although significant progress has been made in multimodal BCI, there is still room for further technological development. For example, as BCI‐FES has successfully regulated hand movements and facial expressions, it may also be feasible to directly act on vocal cord muscles for improved vocalization among dystonia patients. Similarly, LFP‐based brain state decoding techniques have shown feasibility for PD diagnosis [185], suggesting that it may be possible to apply a BCI‐FES system for direct stimulation of efferent nerves and muscles to achieve real‐time mitigation of motor symptoms. However, the realization of this technology relies on high‐precision information recognition and control‐side adjustment, which, in turn, requires further technological breakthroughs and clinical verification.

The typical BCI system is composed of one or more signal recording modules (e.g., EEG electrodes), a signal analysis module (often for EEG spectral analysis), a module for the generation of output instructions (electronic commands), and various execution modules (e.g., robotic arms). In addition, advanced BCI systems are equipped with feedback capability and potential learning algorithms for experience‐dependent improvements in execution. These modules are often designed with separate computation and data storage units; however, this can lead to data transmission bottlenecks, reducing the overall efficiency and energy efficiency of the system. Moreover, current BCI systems are generally too large for true portability, impeding large‐scale clinical applications.

With the rapid development of microelectronics, it has become possible to integrate microprocessors with MEAs on flexible substrate materials [7], to integrate stand‐alone modules into portable devices with wireless communication, and to implant portable wireless devices directly into the human body, such as in the cranial cavity, on the cortical surface, within brain structures, or in the neurovasculature. Similar to the design concept of the brain–neuromorphic interface, which integrates all functions on a single neuromorphic chip [330], it is speculated that this emerging portable BCI architecture can still achieve high‐performance data acquisition, analysis, command generation, and output device control. Moreover, advances in material science have enhanced the biocompatibility of BCI components with brain tissue, allowing for safer and more reliable long‐term function. Through miniaturization, BCI systems are expected to be used at home without reliance on laboratory and hospital environments. For example, users may one day receive signals directly from embedded microprocessors through external signal receivers, mobile phones, or computer applications, making BCI applications truly portable. This integrated design may not only optimize user experience and portability but also reduce healthcare costs while driving technological advancements in related industries.

**FIGURE 6:**
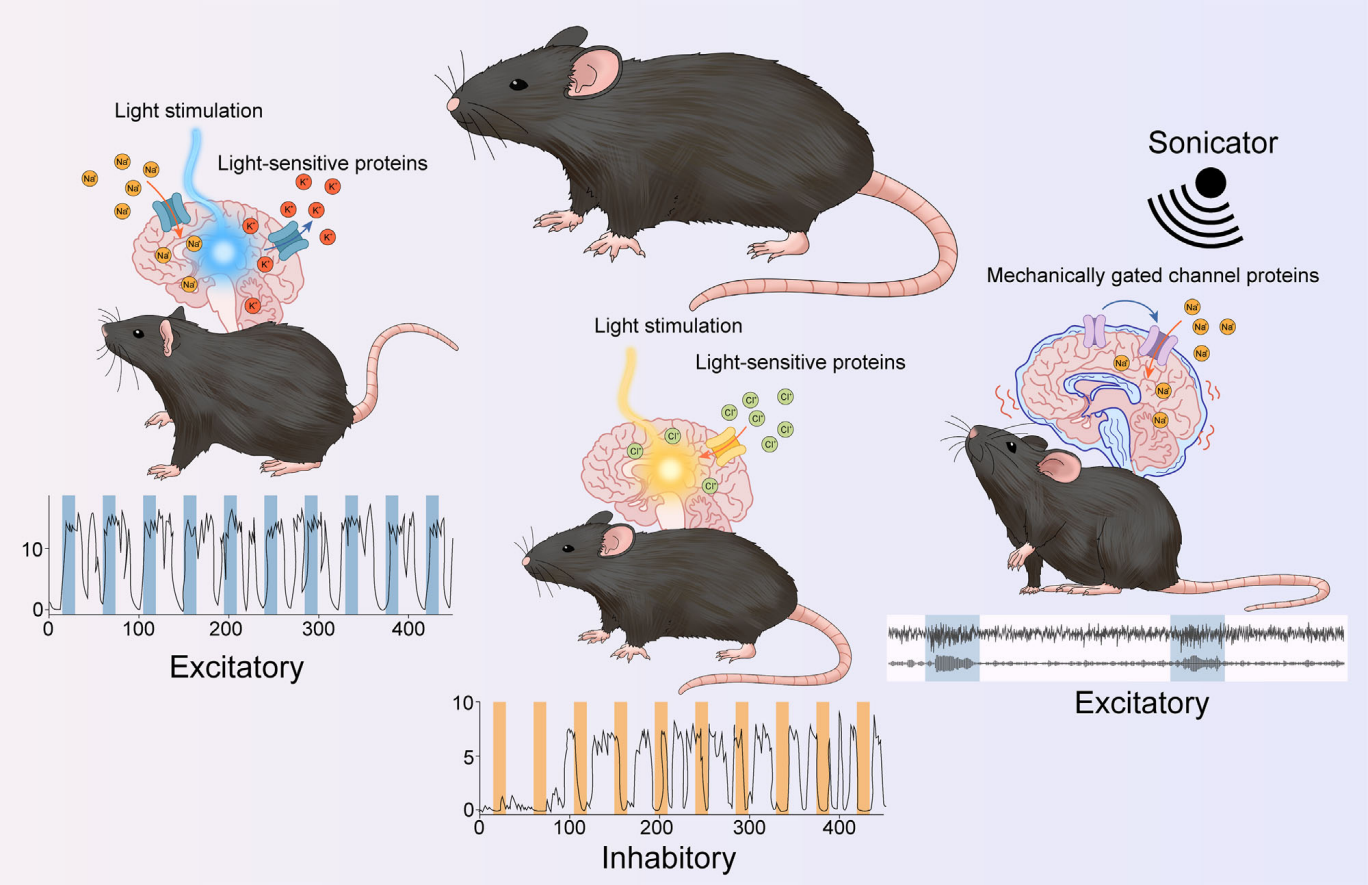
Optogenetics‐ and ultrasound genetics‐based BCI. The schematic diagram illustrates the principles of optogenetics and ultrasonic brain–computer interface systems. In this setup, three mice have been implanted with optical fibers and light sources within their skulls. In two of these mice, light‐sensitive ion channel proteins are expressed in the target neurons of the cerebral cortex. These light‐sensitive ion channels can be selectively activated by different wavelengths of light, thereby generating action potentials or hyperpolarization. Neurons that do not express these channels are not affected by the light stimuli. For example, under blue light stimulation, sodium channels are opened, allowing sodium ions to enter the cell and trigger an action potential, which increases local field potential oscillations. Conversely, when stimulated by yellow light, chloride channels are activated, leading to hyperpolarization and inhibition of neuronal excitation, which reduces local field potential oscillations. Additionally, the mechanism of an ultrasonic‐based brain–computer interface is demonstrated. Mechanosensitive channels are expressed in the target neurons, and specific ultrasonic frequencies induce intracranial cerebrospinal fluid vibrations when applied outside the brain, causing these channels to open. This results in sodium influx, action potential generation, and enhanced local field potential activity.

This integrated model has a variety of potential development directions. For example, the combination of microelectronics technology and optogenetics BCI and the integration of microelectrodes, microprocessors, and miniature light sources for intracranial light stimulation could solve the problem of poor light penetration through the skull and reduce the risk of brain tissue damage by optical fiber implantation. In addition, a portable neurostimulation device has been developed to provide real‐time and accurate FES neuromodulation for patients with movement disorders. These innovative designs not only improve the performance and application range of BCI systems but also provide a new technical path for neuroscience and clinical research. Through the integration and optimization of microelectronic technology, BCI systems are expected to achieve the leap from laboratory to daily life, provide patients with more convenient and efficient treatment options, and promote the continuous development of neuroengineering. The structure of an integrated BCI is depicted in Figure 6.

**FIGURE 7:**
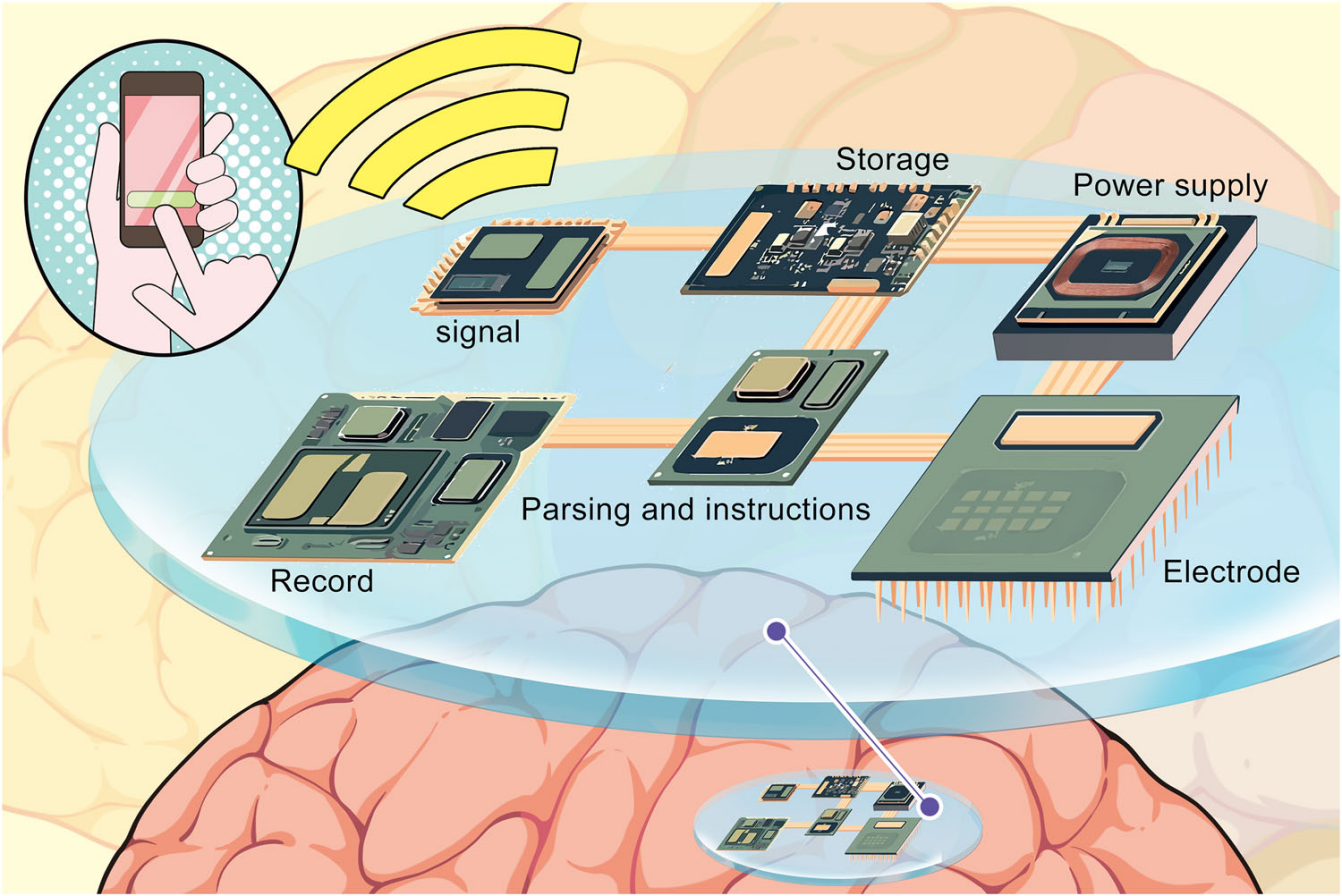
An integrated BCI system. Integrated microelectronic BCI systems may enhance usability for broader clinical application. The cerebral cortex is covered with a flexible substrate, and an integrated module is attached composed of a signal transceiver module, a storage module, a power supply (the power supply is covered with an electromagnetic coil for wireless charging), a microelectrode array, a chip (signal analysis module and command generation module), and an electrical signal recording module (e.g., electrocorticography (ECoG)). The signal transceiver module communicates wirelessly with external devices, such as a mobile phone, and regulates the integrated plate via transceiver instructions such as phone or computer applications. This integrated micro‐electronic BCI architecture is highly efficient, portable, and biocompatible.

Optogenetic BCI has made substantial progress in recent years, but its clinical application still faces many challenges. First, optogenetic stimulation relies on gene editing of light‐sensitive proteins, a process that is not only technically difficult but also ethically controversial. Second, the implantation of optical fibers to transmit light sources may cause mechanical damage to brain tissue, limiting their widespread application in patients. To reduce the risk of brain tissue damage during optical fiber implantation, this paper proposes a novel approach: the induced expression of mechanically gated ion channels in brain tissue activated by ultrasound. Auditory transduction provides a biological example. Hearing depends on the rhythmic fluctuation of lymph fluid in the cochlea driven by sound waves, which in turn causes the vibration of cilia on hair cells and concomitant direction‐dependent opening or closing of mechanically gated potassium channels. The opening of these channels allows potassium influx, which depolarizes the cell and triggers further calcium influx and transmitter release to stimulate the auditory nerve. Based on this principle, it may be possible to induce cerebrospinal fluid vibrations through sound waves of specific frequencies (e.g., ultrasound), so as to activate mechanically gated ion channels expressed in specific neurons and thereby achieve precisely localized excitation. In addition, it may also be possible to integrate the vibration‐generating device with a microelectrode or microprocessor implanted directly in the brain to achieve neuromodulation through the generation and propagation of vibration. This acoustic wave‐based modulation approach has several advantages. First and foremost, sound waves can be transmitted non‐invasively through bone, thus avoiding the potential risk of brain tissue damage by fiber optic implantation. In addition, acoustic control technology can achieve high spatial resolution, and sound can penetrate through tissue to precisely target neurons (acoustic DBS). With further research on mechanically gated ion channels and the optimization of acoustic wave modulation technology, this new neuromodulation strategy could provide a safer and more efficient alternative to optogenetics‐based BCIs. Optogenetics and ultrasound genetics BCI are compared schematically in Figure 7.

The rapid development of AI technology provides new possibilities for the optimization and enhancement of BCI. First, deep learning models have achieved remarkable results in image, speech, and emotion recognition, indicating their applicability to EEG decoding. Second, Bayesian optimization and other algorithms can effectively improve the decoding performance and operational efficiency of BCI systems. Third, CNNs can identify different emotional states, motor patterns, and mental phenotypes, laying the foundation for precise individualized neuromodulation by BCI. Finally, user modeling technology could be integrated into BCI systems to enable personalized adaptation. The deep integration of AI and BCI is expected to provide users with more intelligent, efficient, and personalized treatment while creating additional BCI applications.

The extensive application of AI in BCI remains constrained by algorithmic bias, overfitting, and high computational demands. Algorithmic bias arises from unfairness in training data, model design, or algorithmic implementation, leading to discrimination against specific groups in decision‐making and impairing BCI's fairness and usability [331]; mitigation strategies include embedding ethical codes in model design to introduce fairness constraints and employing cross‐subject decoding methods (e.g., spatio‐temporal convolutional networks with discriminators) based on domain adaptation to enhance general feature representation and reduce performance disparities [332, 333].

Overfitting manifests as strong performance on training data but poor generalization, exacerbated in BCI due to the low SNR and non‐stationarity of EEG signals [334]; solutions involve the CSSBP algorithm integrating RP and ERD features to improve robustness [335], regularization techniques (e.g., L2/L1 regularization [336], SWLDA [332]) to reduce model complexity, and deep learning approaches (e.g., CNN [337]) with adaptive sample weighting to alleviate small‐sample issues [338]. Computational demands pertain to resources required for system operation, necessitating optimization to meet real‐time requirements; strategies encompass lightweight models (e.g., CNN) [339], quantization techniques to reduce computation [340], optimized algorithms [341] (e.g., GA, PSO) for dimensionality reduction and efficiency, and GPU‐based parallel processing or robust parameter estimation (e.g., MDE) to enhance robustness and efficiency [342, 343]. Collectively, these methods aim to advance BCI's fairness, generalization capability, and practicality.

Closed‐loop BCIs leverage adaptive algorithms to personalize neuromodulation, but this raises concerns about overfitting when models are overly tailored to individual patients. To address this trade‐off between model specificity and generalizability, recent advancements in EEG decoding algorithms have introduced robust strategies. For instance, regularized methods such as L1‐regularized multiway CCA and shrinkage linear discriminant analysis mitigate overfitting by constraining model complexity while preserving individual‐specific features [334, 335, 336, 337, 338, 339, 340, 341, 342, 343, 344]. Additionally, Riemannian geometry‐based approaches, which exploit the intrinsic curvature of covariance matrices, enhance generalization by aligning with the natural structure of EEG data [334]. These methods are particularly critical for disorders like depression, where symptom heterogeneity necessitates balancing personalized feedback with population‐level adaptability. For example, dynamic adjustment of stimulation parameters based on real‐time neural feedback, as demonstrated in depression treatment studies, requires algorithms that generalize across diverse patient profiles [345]. Furthermore, literature emphasizes the need to caution against excessive personalization risks, advocating systematic approaches over reliance on clinical features in the absence of clear predictive indicators [346, 347]. Consequently, algorithm design should synthesize adaptive mechanisms with cross‐patient generalization frameworks to harmonize individualized needs and population adaptability. Future research should further integrate multimodal data and transfer learning to bridge the gap between individualized and scalable BCI systems [65].

The integration of EEG and fNIRS in BCIs leverages their complementary temporal and spatial resolutions, yet challenges arise from temporal synchronization and signal latency discrepancies. To address these issues, computational strategies such as feature‐level fusion (e.g., normalized features or multi‐domain feature extraction) and decision‐level fusion (e.g., combining classifier outputs) have been proposed to harmonize multimodal signals [348, 349]. For instance, studies have demonstrated that aligning EEG and fNIRS features using multi‐level progressive learning frameworks can mitigate fNIRS's hemodynamic delay, enabling real‐time decoding of motor tasks [349, 350]. Additionally, hybrid BCI architectures, such as sequential or parallel processing systems, allow for temporal alignment by prioritizing EEG's high temporal resolution while compensating for fNIRS's spatial advantages [348, 351]. Future research should focus on optimizing these strategies to enhance ecological validity and computational efficiency, particularly in low‐latency applications [349, 352].

The significant intra‐ and inter‐subject variability inherent in neural signals presents substantial challenges for BCI algorithms. While current AI‐driven neural decoders, such as CNNs and LSTMs, demonstrate remarkable performance in EEG signal processing, their efficacy in managing neural signal non‐stationarity and individual variability remains a critical limitation. To address this, contemporary approaches integrate adaptive decoding frameworks, including online parameter updates and unsupervised learning mechanisms like non‐stationary modeling based on random walk assumptions, alongside feedback mechanisms such as error‐driven signal adjustment. These techniques facilitate adaptation to individual neurophysiological “fingerprints” without necessitating extensive recalibration [353, 354]. Architectures like recurrent neural networks, for instance, convolutional LSTMs, retain temporal dynamics through sequential modeling, while other deep learning architectures leverage noise robustness to accommodate inter‐subject differences [355, 356]. Furthermore, recent studies have explored adaptive frameworks leveraging transfer learning and subject‐independent feature extraction. For example, Riemannian geometry‐based classifiers, such as the minimum distance to Riemannian mean algorithm, exploit the invariance of covariance matrices to mitigate inter‐subject variability [357]. Additionally, deep learning models incorporating attention mechanisms and multi‐task learning paradigms show promise in enhancing generalization across diverse neural patterns [358, 359]. These methodological advancements not only reduce the need for extensive recalibration but also align with the growing demand for user‐independent BCI systems. Future research should prioritize the further integration of unsupervised domain adaptation techniques to enhance the robustness of these technologies in real‐world applications.

Nanomaterials can be fabricated with a variety of characteristics advantageous for BCI, including high flexibility, hypoallergenicity, physical and chemical stability, and malleability, all of which contribute to reducing the risk of mechanical and inflammatory brain tissue damage. In addition, carbon nanomaterials can be constructed into scaffolds with luminal structures containing factors or cells that promote brain tissue repair following implantation [360] or therapeutic drugs for stimulus‐dependent release. For instance, luminal structures can be used to carry anti‐inflammatory factors that are continuously released to reduce the accumulation of inflammatory mediators and prolong the service life of the electrode. In fact, any material with a rich cavity structure and sufficient conductivity, stability, and malleability could be used to fabricate drug‐delivery or repair‐promoting electrodes. Magnetoelectric nanomaterials are also promising materials for the fabrication of DBS electrodes, as magnetic exposure has several advantages over FES for certain applications.

Future developments should also focus on multifunctional BCI electrodes suitable not only for electrical stimulation but also for neurochemical measures, disease monitoring, neuromodulation, and pharmacological treatment. Carbon nanoelectrodes can be fabricated for drug release and biomolecule monitoring, underscoring the advantages of selecting highly versatile electrode materials. Such electrodes may be especially valuable for the monitoring and treatment of PD. For instance, carbon nanoelectrodes could be implanted into the substantia nigra for stimulation and for monitoring dopamine levels as a trigger for releasing anticholinergic drugs or levodopa as needed. For some diseases where the neurobiological mechanism is not yet clear, such as autism, this type of invasive electrode is expected to provide a means for clarifying pathogenesis by detecting biomolecular changes in the brain.

In conclusion, future research on BCIs should prioritize multimodal integration and AI‐driven personalization. For instance, the integration of EEG, fMRI, and optogenetics can enable spatiotemporally precise neuromodulation. Integrating multiple BCI functions onto a single chip can enhance portability, while AI algorithms, such as CNNs, can optimize the real‐time decoding of individual neural patterns. Furthermore, ultrasonic genetics offers a non‐invasive neuromodulation strategy by activating mechanically gated channels, thereby overcoming the invasive risks associated with optogenetic BCIs. These advancements are poised to drive the development of BCI systems toward adaptive, wireless, and fully implantable platforms for chronic disease management. By advancing these research directions and addressing associated challenges, BCI technology is expected to achieve breakthroughs in broader clinical applications, including the treatment of chronic pain; restoration of lost motor, sensory, and communication functions; diagnosis of neuropsychiatric disorders; event‐triggered interventions; and rehabilitation. These advances will also foster innovation in emerging fields such as VR, AR and broader forms of human–computer interaction, potentially reshaping daily life.

The realization of this vision depends critically on concurrent advances in several foundational domains. The rapid development of materials science has provided novel material options for the design and enhancement of BCI electrodes, such as carbon nanomaterials and biomaterials, which can improve signal quality, mitigate biocompatibility issues, and enhance long‐term stability, thereby propelling BCI technology toward safer and more reliable applications. The selection of appropriate animal models is essential for the preclinical validation of BCI technologies; for example, genetically modified mice and transgenic mice can simulate diverse human disease pathologies, enabling the verification of BCI efficacy and the assessment of safety, thus providing crucial evidence for clinical translation. Clinical translation represents a pivotal step in the practical application of BCI technology, requiring the resolution of challenges related to technological maturity, regulatory approval, patient acceptance, and long‐term efficacy assessment. Through multidisciplinary collaboration among neuroengineers, clinicians, ethicists, and policymakers, the healthy development of BCI technology can be promoted, and an ecosystem supporting its clinical translation can be established. Such concerted efforts are expected to facilitate significant breakthroughs in BCI technology, offering novel possibilities for the treatment of neurological and psychiatric disorders. In summary, the potential of BCIs is immense, limited only by the bounds of our current imagination.

## Nomenclature

aBCI: acoustic brain–computer interface
AD: Alzheimer's disease
aDBS: adaptive deep brain stimulation
ADHD: attention‐deficit hyperactivity disorder
ADHD‐RS: Attention Deficit Hyperactivity Disorder Rating Scale
AI: artificial intelligence
ALS: amyotrophic lateral sclerosis
AMD: age‐related macular degeneration
AOT: action observation therapy
AR: augmented reality
ARAT: action research arm test
AROM‐WE: active range of motion in wrist extension
ASD: autism spectrum disorder
BCI: brain–computer interface
BCI‐FES: brain–computer interface‐functional electrical stimulation
BD: bipolar disorder
BMI: brain–machine interface
CCA: canonical correlation analysis
cDBS: conventional deep brain stimulation
CLIS: complete locked‐in syndrome
cNF: cognitive neurofeedback
CNN: convolutional neural network
CNTs: carbon nanotubes
CSP: common spatial patterns
CST: cortical‐spinal tract
DBS: deep brain stimulation
DCS: direct current stimulation
DOC: disorders of consciousness
ECoG: electrocorticography
ECT: electroconvulsive therapy
EEG: electroencephalography
EGaIn: eutectic gallium‐indium
EMG: electromyography
EPN‐DBS: entopeduncular nucleus‐deep brain stimulation
ERD: event‐related desynchronization
ERN: error‐related negativity
ERPs: event‐related potentials
ErrP: error‐related potential
ETDRS: early treatment diabetic retinopathy study
FER: facial emotion recognition
FES: functional electrical stimulation
FFA: fusiform face area
FMA‐UE: Fugl–Meyer assessment for upper extremity
fMRI: functional magnetic resonance imaging
fNIRS: functional near‐infrared spectroscopy
FSS: frequency‐specific synchronization
GA: genetic algorithm
GPi: globus pallidus
HGE: high gamma energy
iBCI: intracortical brain–computer interface
ICA: independent component analysis
iEEG: intracranial electroencephalography
kNN: k‐nearest neighbor
LDA: linear discriminant analysis
LFP: local field potential
LIS: locked‐in syndrome
logMAR: logarithm of the minimum angle of resolution
LSTM RNN: long short‐term memory recurrent neural network
MAL: motor activity log
MBI: modified Barthel Index
MCS: minimally conscious state
MDD: major depressive disorder
MEA: microelectrode array
mECoG: multi‐channel electrocorticography
MEG: magnetoencephalography
MENDs: magnetoelectric nanodisks
MEP: motor‐evoked potential
MI: motor imagery
MI‐BCI: motor imagery brain–computer interface
MoCA: Montreal Cognitive Assessment
MRCP: movement‐related cortical potential
MRC‐WE: Medical Research Council Wrist Extensor
MRI: magnetic resonance imaging
MVPA: multivariate pattern analysis
NF: neurofeedback
NMD: neuromuscular disease
OB: olfactory bulb
OHDA: 6‐hydroxydopamine
P300: P300 event‐related potential
PD: Parkinson's disease
PICK: poststroke cognitive impairment
PRIMA: photovoltaic retinal implant for macular atrophy
PTSD: post‐traumatic stress disorder
RCT: randomized controlled trial
RF: random forest
RNS: responsive neurostimulation
rTMS: repetitive transcranial magnetic stimulation
SAEs: serious adverse events
SCI: spinal cord injury
SCP: slow cortical potential
sEEG: stereoelectroencephalography
sEMG: surface electromyography
SNR: signal‐to‐noise ratio
SSVEP: steady‐state visual evoked potential
STN: subthalamic nucleus
STN‐DBS: subthalamic nucleus deep brain stimulation
STN‐LFP: subthalamic local field potentials
SVM: support vector machine
SVZ: subventricular zone
tACS: transcranial alternating current stimulation
TBS: theta burst stimulation
TD: typically developing
tDCS: transcranial direct current stimulation
TES: transcranial electrical stimulation
TMS: transcranial magnetic stimulation
TRD: treatment‐resistant depression
UWS: unresponsive wakefulness syndrome
V1: primary visual cortex
VR: virtual reality
VS: vegetative state
VT2: vibrotactile with two stimuli
VT3: vibrotactile with three stimuli
WMFT: Wolf Motor Function Test
XGBoost: extreme gradient boosting
XR: extended reality

## Author Contributions

Yuqi Feng, Wangzheqi Zhang, Chenglong Zhu, Yisheng Chen, and Lei Wu contributed to the manuscript writing and figure preparation; Jun Chen, Huang Wu, and Yanhao Qiu designed the work; Xiaomin Zhang, Zhijie Zhao, Changli Wang, and Xiaoming Deng supervised the work. All authors have read and approved the article. All authors read and approved the final manuscript.

## Funding

This study was funded by the Fund Project of National Natural Science Foundation of China (82302421) and the Fund Project of National Natural Science Foundation of China (82272214).

## Ethics Statement

This manuscript does not contain any studies with human participants or animals performed by any of the authors.

## Conflicts of Interest

The authors declare no conflicts of interest.

## Data Availability

Data sharing is not applicable to this article as no datasets were generated or analyzed during the current study. All information is derived from publicly available articles and datasets.
